# Patterns in floral traits and plant breeding systems on Southern Ocean Islands

**DOI:** 10.1093/aobpla/plv095

**Published:** 2015-08-18

**Authors:** Janice M. Lord

**Affiliations:** Botany Department, University of Otago, PO Box 56, Dunedin 9054, New Zealand

**Keywords:** Cool temperate, dioecy, gynodioecy, self-incompatibility, southern hemisphere, subantarctic, wind-pollination

## Abstract

The harsh climatic conditions and paucity of potential pollinators on Southern Ocean Islands (SOIs; latitude 46°S–55°S) lead to the expectation that anemophily or self-fertilization are the dominant modes of plant sexual reproduction. However, at least some species have showy inflorescences suggesting biotic pollination or dimorphic breeding systems necessitating cross-pollination. This study investigates whether anemophily and self-compatibility are common on SOIs, whether species or genera with these traits are more widespread or frequent at higher latitudes, and whether gender dimorphy is correlated with anemophily, as might occur if reliance on pollinators was a disadvantage. Of the 321 flowering plant species in the SOI region, 34.3 % possessed floral traits consistent with anemophily. Compatibility information was located for 94 potentially self-fertilizing species, of which 92.6 % were recorded as partially or fully self-compatible. Dioecy occurred in 7.1 % of species overall and up to 10.2 % of island floras, but has not clearly arisen *in situ*. Gynodioecy occurred in 3.4 % of species. The frequency of anemophily and gender dimorphy did not differ between the SOI flora and southern hemisphere temperate reference floras. At the species level, gender dimorphy was positively associated with fleshy fruit, but at the genus level it was associated with occurrence in New Zealand and a reduced regional distribution. Anemophily was more prevalent in genera occurring on subantarctic islands and the proportion of species with floral traits suggestive of biotic pollination was significantly higher on climatically milder, cool temperate islands. These results support the contention that reliance on biotic pollinators has constrained the distribution of species on SOIs; however, it is also clear that the reproductive biology of few SOI species has been studied *in situ* and many species likely employ a mixed mating strategy combining biotic pollination with self-fertilization.

## Abstract (precis)

Little is known about floral biology in the Southern Ocean region 45° - 55°S, despite the World Heritage status of some areas. Typical pollinators are rare on these cold, remote islands, yet some plants have showy flowers, suggesting insect pollination, or separate sexed flowers requiring cross-pollination. This study analyses data for 321 species on 11 Southern Ocean island groups. Separate sexed flowers are common compared with other high latitude islands, but a reliance on biotic pollination may limit species distributions. Given the vulnerability and uniqueness of these floras, a greater effort needs to be made to understand their reproductive ecology.

## Introduction

Isolated islands provide an unparalleled opportunity to study evolutionary processes in plants (Carlquist 1977; Lloyd 1985; Sakai *et al*. 1995; Grant 1998; Losos and Ricklefs 2009). In particular, the study of island floras has made a significant contribution to our understanding of the evolution of plant reproductive strategies, as islands often lack guilds of pollinators common on larger landmasses and new colonists can be further disadvantaged by small population sizes, increasing the risk of inbreeding depression (Carlquist 1977; Grant 1998). Compared with mainland relatives, island plants tend to have smaller, less brightly coloured floral displays, lower flower visitation rates and a greater incidence of anemophily (wind pollination) (Carlquist 1977; Inoue *et al*. 1995; Barrett 1998). Self-compatible hermaphrodites, capable of producing seed via self-fertilization, should have an advantage in establishing on isolated islands (Baker 1955). Selection should also act strongly in favour of the evolution of self-compatibility in biotically pollinated, out-crossing taxa if pollinators are scarce (Barrett 1998). However, islands are also associated with a high incidence of dioecy (Carlquist 1977; Bawa 1982; Sakai *et al*. 1995; Barrett 1998; Webb *et al*. 1999; see Table 1 for breeding system terminologies), possibly due to selection for mechanisms that reduce the likelihood of inbreeding depression, as well as the well-known correlation between dioecy and fleshy fruits, which could make long-distance dispersal more likely (Bawa 1982; Sakai *et al*. 1995; Sakai and Weller 1999; Webb *et al*. 1999). Baker and Cox (1984) further linked the frequency of dioecy in island floras to moist tropical climates and the probable source flora, but highlighted the paucity of the available data. Despite the importance of islands in understanding these evolutionary processes in plants, plant reproductive traits have still been investigated in remarkably few island floras and complete data are still lacking even for well-studied islands (Bernardello *et al*. 2001; Newstrom and Robertson 2005; Chamorro *et al*. 2012).

**Table 1.** An overview of flowering plant breeding system terminologies and the frequency of selected breeding systems worldwide (from Richards 1997; Renner 2014).

System	Description	Frequency
Self-compatible	Pollination of a receptive stigma by pollen from the same plant leads to viable seed.	∼61 % of species
Self-incompatible	Pollination of a receptive stigma by pollen from the same plant (or another plant carrying the same genetic recognition factors) does not lead to viable seed.	∼39 % of species
Hermaphrodite	Plants monomorphic. All flowers on an individual plant are functionally male and female (i.e. cosexual). Plants can potentially self-pollinate by pollen transfer to a receptive stigma within or between flowers.	∼72 % of species
Monoecy	Monomorphic. Flowers on an individual plant are either male or female. Plants can potentially self-pollinate by pollen transfer from male flowers to female flowers.	5–6 % of species
Gynomonoecy	Monomorphic. Flowers on an individual plant are either cosexual or female. Plants can potentially self-pollinate either by pollen transfer within cosexual flowers or transfer of pollen from cosexual to female flowers.	∼2.8 % of species, common in Asteraceae
Andromonoecy	Monomorphic. Flowers on an individual plant are either cosexual or male. Plants can potentially self-pollinate by pollen transfer within cosexual flowers.	∼1.5 % of species
Dioecy	Dimorphic. Plants are either entirely female or entirely male. Plants cannot self-pollinate.	5–6 % of species
Gynodioecy	Dimorphic. Plants are either entirely female or entirely hermaphrodite. Females cannot self-pollinate. Hermaphrodites can potentially self-pollinate.	∼7 % of species

The Southern Ocean is home to numerous island groups which have received relatively little attention from island biologists. This region is here defined following Greve *et al*. (2005) as including the southern waters influenced by westerly winds as well as the subantarctic region influenced by the Antarctic Convergence. Southern Ocean Islands (SOIs) between latitude 46°S and 55°S have in common their relative isolation, cold oceanic climate and a dominance of herbaceous and graminoid vascular plants. Islands in the region vary in size from <3 to >3000 km^2^ and vascular plant species richness relates strongly to island area as well as sea surface temperature (Chown *et al*. 1998). The floras of SOIs have derived substantially from survivors of a native subantarctic flora, rather than post-glacial maximum long-distance dispersal colonists (Wagstaff and Hennion 2007; Van der Putten *et al*. 2010; Wagstaff *et al*. 2011). However, long-distance dispersal from larger landmasses to the north such as Australia, New Zealand and South America, as well as within and among SOI groups, has produced strong patterns of nestedness and regionalization (Chown *et al*. 1998; Greve *et al*. 2005; Van der Putten *et al*. 2010).

With the exception of the Falklands Islands (Islas Malvinas), the islands of the Southern Ocean lack butterflies and bees (Gressitt 1964; Lloyd 1985; Donovan 2007; Schermann-Legionnet *et al*. 2007; Convey *et al*. 2010). Furthermore, the climate is characterized by high winds and low temperatures which would appear unsuitable for flying insects; in fact flightlessness has evolved in many SOI insect groups (Gressitt 1964; Chown *et al*. 1998). This has led to the suggestion that plants on SOIs mainly rely on anemophily (pollen transfer via wind) or self-fertilization, rather than biotic pollination, in order to reproduce sexually (Smith 1984; Bergstrom *et al*. 1997; Schermann-Legionnet *et al*. 2007; Convey *et al*. 2010). On some SOIs, however, dioecious species, as well as species with showy floral displays, are a conspicuous component of the flora. If the depauperate insect fauna and harsh climatic conditions on SOIs have selected against reliance on biotic pollination, it might be that species with showy flowers, while conspicuous, are atypical and, further, that dimorphic breeding systems are successful only in conjunction with anemophily. Experimental studies of SOI plant breeding systems and pollination modes have produced a range of results. Bergstrom *et al*. (1997) demonstrated autogamous self-compatibility or suspected facultative cleistogamy in seven species on Macquarie Island and Schermann-Legionnet *et al*. (2007) described effective self-pollination in *Pringlea antiscorbutica* (Brassicaceae) on Îles Kerguelen. However, Nicholls (2000) and Lord *et al*. (2013) found varying degrees of reliance on biotic pollination on Campbell Island, including self-incompatibility in two species.

The aim of this study was to examine the frequency and distribution of reproductive traits among native flowering plants on SOIs. In particular, I tested whether self-compatibility and anemophily are common in the SOI region compared with mainland floras at similar latitudes, whether anemophilous and self-compatible species occur on more SOIs, as might be expected if such species encountered fewer barriers to establishment and whether species with floral traits suggestive of biotic pollination are restricted to islands with milder climates. I also tested whether gender dimorphic breeding systems (dioecy and gynodioecy) are particularly uncommon, associated with anemophily, or show taxonomic or biogeographic associations with the New Zealand flora, which has a high incidence of gender dimorphism (Webb *et al*. 1999).

## Methods

### Islands included

The 11 SOI groups used in this study include all of the islands mentioned in Van der Putten *et al*. (2010) and all of the island groups south of latitude 46°S used by Chown *et al*. (1998) and Greve *et al*. (2005) with the exception of the Bounty Islands. The Bounty Islands are not included as only one flowering plant species has been described from the island group (Amey *et al*. 2007; de Lange *et al*. 2013), and it has not been relocated recently (J. Hiscock, New Zealand Department of Conservation, pers. comm.). Following Smith (1984), the climate zone of the Snares, Antipodes, Auckland, Campbell and Falklands Islands was classed as cool temperate and that of Prince Edward & Marion Islands, Îles Crozet, Îles Kerguelen, Heard and MacDonald, South Georgia and Macquarie Islands was classed as subantarctic. Data on total island group land area, median latitude and longitude and phytogeographic province were obtained from Chown *et al*. (1998) and Van der Putten *et al*. (2010).

### Reference floras

Reference floras from New Zealand, Stewart Island, Tierra del Fuego and alpine Patagonia were used for statistical analysis of SOI flora composition. These areas are relevant to the SOI flora as they extend beyond latitude 45°S, include cool temperate forest and alpine vegetation and encompass the South Pacific and South Atlantic phytogeographic provinces of Van der Putten *et al*. (2010). No mainland reference flora was available for the SOI South Indian province identified in Van der Putten *et al*. (2010) as no large landmass extends below 35°S in the Indian Ocean. Stewart Island (∼47°S, 168°E; 1720 km^2^) lies 27 km south of mainland New Zealand, to which it was connected during the last glacial maximum. A list of native flowering plant species was obtained from Wilson (1982), and floral traits and breeding systems were extracted from Allan (1961), Moore and Edgar (1970), Webb *et al*. (1988) and Edgar and Connor (2000). The frequency of self-compatibility in native New Zealand flowering plants was obtained from Newstrom and Robertson (2005). Breeding system, pollination mode and self-compatibility information for the native flowering plant flora of alpine Chilean Patagonia (∼50°S, 73°W) were obtained from Arroyo and Squeo (1990). The flora of Tierra del Fuego (∼54°S, 69°W; Moore 1983) was used as an additional reference to determine whether particular families were over represented in the SOI flora.

### The SOI flora

Data on SOI plant species occurrences and reproductive traits were obtained from Greene (1964), Moore (1968), Huntly (1971), Johnson and Campbell (1975), Meurk (1975), Godley (1989), Chown *et al*. (1998), Hay *et al*. (2004), Broughton and McAdam (2005), Upson (2012) and the Flora of New Zealand series (Allan 1961; Moore and Edgar 1970; Webb *et al*. 1988; Edgar and Connor 2000), which includes all SOIs in the Pacific province (Table 2). The complete SOI flora used in this study consisted of 321 flowering plant species in 51 families and 150 genera, of which 81 species (25.2 %) were endemic to the region. The majority of species (62.5 %) were eudicots and the majority of genera (57.7 %) were represented by a single species; only 20 genera (13.3 %) contained four or more species. Single island occurrences constituted the majority of records (64.7 %); only 15 species occurred on five or more islands. The largest genera in the SOI region are *Carex* (17 species), *Poa* (15) and *Ranunculus* (11), and the largest families are Poaceae (47 species), Asteraceae (46), Cyperaceae (35), Apiaceae (21, including *Stilbocarpa* following Mitchell *et al*. 1999) and Orchidaceae (18). The relative contributions to the SOI flora of these families, and the fifth category, ‘others’, were similar to the Stewart Island flora (*χ*^2^ = 4.46, *P* > 0.4, df = 5), but differed from both the alpine flora of Patagonia and the flora of Tierra del Fuego (*χ*^2^ = 22.69, *P* < 0.001, df = 5; *χ*^2^ = 15.43, *P* < 0.01, df = 5, respectively). In both of the latter cases, the SOI flora had significantly more orchid species (cell *χ*^2^ values >5), but did not differ in the relative contributions of the other five categories.

**Table 2.** Features of Southern Ocean Islands and their flowering plant floras. Province follows Van der Putten *et al*. (2010). SC, fully or partially self-compatible species; Comp. known, species for which information on the ability to self-fertilize was located.

Island group	Median Lat. (°S)	Median Long.	Area (km^2^)	Province	Total species	Dioecious/gynodioecious	SC species/comp. known
Îles Crozet	46.24	51.22°E	356	Indian	17	0/1	9/9
Prince Edward & Marion Islands	46.77	37.35°E	334	Indian	15	0/1	8/8
The Snares	48.12	166.6°E	3	Pacific	13	1/0	1/1
Îles Kerguelen	49.37	69.5°E	7200	Indian	22	0/1	11/11
Antipodes Islands	49.7	178.8°E	21	Pacific	49	5/0	11/12
Auckland Islands	50.83	166.0°E	626	Pacific	138	11/1	18/21
Falklands Islands	51.5	59.50°W	8500	Atlantic	151	9/9	63/67
Campbell Island	52.5	169.2°E	113	Pacific	99	10/1	18/21
Heard & MacDonald Islands	53.07	73.05°E	371	Indian	11	0/1	4/4
South Georgia	54.25	37.0°W	3755	Atlantic	16	0/1	9/9
Macquarie Island	54.62	158.9°E	128	Pacific	36	2/1	10/11

### Sources of species trait information

All species names were cross-checked for synonymy, taxonomical validity and authority using the online taxonomic resource The Plant List (2013). Further information on flora composition, floral traits and breeding systems was obtained via keyword searches on Web of Science, Google Scholar and internet search engines for journal articles, reports and dissertations, using genus, species and island names as search terms. British Antarctic Survey Bulletins and publications were also searched for relevant information. For some non-endemic species, information could only be obtained from species descriptions in the floras of Tierra del Fuego (Moore 1983) and Patagonia (Arroyo and Squeo 1990; Arroyo *et al*. 1992). Taxonomic treatments of SOI genera were also examined for statements concerning breeding system characteristics of species and subgenera, as well as statements concerning the uniformity of breeding systems within genera.

### Data analysis

Species were assigned one of four floral types based on descriptions of reproductive structures in the literature. Floral type ‘A’ (FTa) lacked sterile display structures and possessed long-exserted anthers and stigmas, consistent with anemophily. Floral type ‘B’ (FTb) showed investment in conspicuous, usually coloured petals or other sterile display structures, potentially indicating signalling to animal pollinators. Species with inconspicuous petals but lacking exserted sex organs were classed as ambiguous (FTab). Species with very small (<2 mm diameter), solitary flowers lacking exserted sex organs may rely on self-fertilization, but as direct evidence of this was generally lacking, such species were classified as ‘minute’ (FTm). Where variability in breeding system was mentioned for a species, the most commonly occurring breeding system was used in analyses. For SOI endemic species, the information obtained generally did not mention variation among island populations. All species traits and sources of information are listed in **Supporting Information—Table S1**.

*χ*^2^ Tests of independence were used to determine taxonomic bias in the SOI flora, and bias in the frequency of gender dimorphic breeding systems, self-compatibility and wind pollination compared with the reference floras. All *χ*^2^ analyses were performed using Statistix v.9 (Analytical Software).

Generalized linear models (GLMs) with a binary error distribution and a logit link function were used to test explanatory relationships between self-compatibility, floral type, gender dimorphy, island occurrence and taxonomic affinity with the New Zealand flora. The low level of endemicity and lack of species-rich lineages suggest that most SOI species have dispersed to, or among, SOIs independently. Thus individual species can be treated as separate tests of the ecological association between a trait and the ability to establish on an island (Westoby *et al*. 1995). From the limited experimental data available even closely related endemic species can differ in reproductive traits (e.g. *Pleurophyllum*, Lord *et al*. 2013). Initial analyses used all 321 species and treated floral type, dimorphic breeding system and self-compatibility as binary response variables (1 for presence, 0 for absence). In order to test the leverage of larger genera in which trait correlations may be shared due to niche conservatism (Lord *et al*. 1995), analyses were repeated at the level of genus. The prevalence of traits among island floras was also analysed in relation to the island attributes latitude, climate zone and phytogeographic province, with an additional factor allowing for the possibility that increased representation of uniformly anemophilous taxa such as Poaceae and Cyperaceae may bias the model. Analyses involving genera and islands treated the number of species within a genus or on an island as ‘trials’ and the number of species exhibiting a particular floral type or a gender dimorphic breeding system as ‘events’. Optimal models were determined using backwards stepwise regression, with the inclusion of a predictor variable dependent on the significance of Type III MS and Wald *χ*^2^ values. All regressions were performed in SPSS Statistics V. 22 (IBM Corporation).

## Results

### Self-compatibility

**Figure 1.**
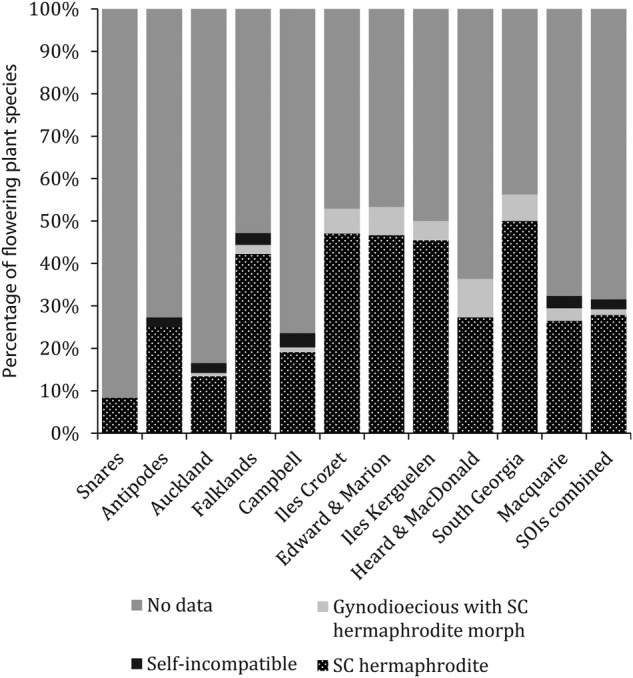
The frequency of known obligate outcrossing (self-incompatible or dioecious) species, fully or partially self-compatible species and species lacking compatibility information for flowering plants native to 11 Southern Ocean Islands. SC, self-compatible. The five island groups clustered on the left side of the horizontal axis are cool temperate. The six island groups clustered to the right of the horizontal axis are subantarctic. Island groups within each cluster are ordered by latitude.

Compatibility information was located for 31.5 % (94 species) of the 298 SOI species that were potentially capable of at least partial self-fertilization (dioecious species excluded) and included 89 monomorphic species and the hermaphroditic morphs of five gynodioecious species. Full or partial self-compatibility has been reported for 83 of these 94 species (92.6 %), including the hermaphroditic morphs of four gynodioecious species. Compatibility information derived directly from SOIs was located for 71 of these species (23.8 % of potentially self-fertilizing species). For the remaining 23 species (all non-SOI endemics), information was extracted from species descriptions or studies from mainland areas. Of the 71 species for which SOI-specific information was available, 95.8 % were fully or partially self-compatible. The GLM found no relationship between self-compatibility and species distribution among islands, climate zones and biogeographic provinces (Fig. 1, Tables 2 and 3).

**Table 3.** Regression models for relationships among gender dimorphy, floral traits, distribution and taxonomic affinities of species, genera and islands. CZIS, binary, island in (1) subantarctic climate zone, (0) cool temperate climate zone; CZOCC, categoric; 0, only in cool temperate climate zone; 1, in both climate zones; 2, only in subantarctic climate zone; FTa, binary; 1, anemophilous floral traits; 0, other types; FTb, binary; 1, floral traits suggesting biotic pollination; 0, other types; FF, binary; 1, fleshy fruit; 0, dry fruit; LAT, island latitude in degrees; NZG, binary; 1, genus present in New Zealand; 0, absent; ALT/IND/PAC: binary, 1, species or genus in Atlantic, Indian or Pacific provinces as defined by Van der Putten *et al*. (2010); 0, absent; PROV, categoric, province; TOTIS, number of islands on which a species or genus occurs; TOTPROV, number of provinces in which a species or genus occurs; df, degrees of freedom. Model *χ*^2^ = Omnibus *χ*^2^ test of model significance, df = 1.

Dataset	Response variable	Predictors tested	df	Significant predictors	Coefficient	Wald *χ*^2^ (*P* value)	Model *χ*^2^ (*P* value)
All species (*N* = 321)	FTa (0,1)	ATL	1				
		CZOCC	2				
		IND	1				
		NZG	1	NZG	2.324	14.601 (0.000)	26.372 (0.000)
		PAC	1				
		TOTIS	1				
		TOTPROV	1				
All species (*N* = 321)	Dimorphic (0,1)	ATL	1				
		CZOCC	2				
		FTa	1				
		FF	1	FF	2.775	36.957 (0.000)	34.778 (0.000)
		IND	1				
		NZG	1				
		PAC	1				
		TOTIS	1				
		TOTPROV	1				
Comp. known (*N* = 94)	Self-compatible (0,1)	ATL	1				
		CZOCC	2				
		IND	1				
		NZG	1	None			
		PAC	1				
		TOTIS	1				
		TOTPROV	1				
149 Genera	Number of species with FTa = 1	ATL	1				
		CZOCC	2	CZOCC-1	0.908	11.679 (0.001)	
				CZOCC-2	1.358	1.124 (0.289)	
		IND	1				34.349 (0.000)
		NZG	1				
		PAC	1	PAC	1.144	7.242 (0.007)	
		TOTIS	1				
		TOTPROV	1				
149 Genera	Number of gender dimorphic species	ATL	1				
		CZOCC	2				
		IND	1				
		NZG	1	NZG	6.187	30.136 (0.000)	107.221 (0.000)
		PAC	1	PAC	2.235	4.221 (0.040)	
		TOTIS	1				
		TOTPROV	1	TOTPROV	−1.536	12.754 (0.000)	
11 Islands	Number of gender dimorphic species	LAT	1				
		PROV	1	None			
		CZIS	1				
11 Islands	Number of species with FTa = 1	LAT	1				
		PROV	1	None			
		CZIS	1				
11 Islands	Number of species with FTb = 1	LAT	1				
		PROV	1				17.842 (0.000)
		CZIS	1	CZIS	−0.931	16.433 (0.000)

### Floral types

**Figure 2.**
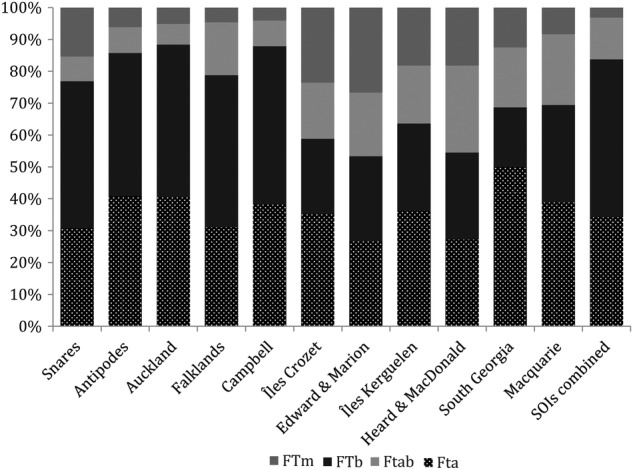
The frequency of floral types among flowering plants native to 11 Southern Ocean Island groups. Arrangement of island groups along the horizontal axis follows Figure 1. FTa, floral traits consistent with anemophily; FTb, flora traits suggestive of biotic pollination; FTab, ambiguous anemophilous or biotically pollinated flowers; FTm, minute flowers.

For the SOI flora as a whole, FTa species, possessing floral traits consistent with anemophily, were less common (34.3 %) than FTb species, possessing floral traits suggestive of biotic pollination (49.5 %). Floral type ‘A’ species, as opposed to other floral types combined, were no more common in the SOI flora than in the floras of Stewart Island (*χ*^2^ = 0.28, *P* > 0.5, df = 1) or alpine Patagonia (*χ*^2^ = 0.88, *P* > 0.5, df = 1). Generalized linear models found that FTa species were more common in genera that also occurred in New Zealand, and were more common in genera that occurred in the Pacific Province and on subantarctic islands (Table 3). Neither latitude, climate zone nor the prevalence of Poaceae and Cyperaceae explained the prevalence of FTa species on islands, but FTb species were more common on cool temperate as opposed to subantarctic islands (Fig. 2, Table 3).

### Breeding systems

**Figure 3.**
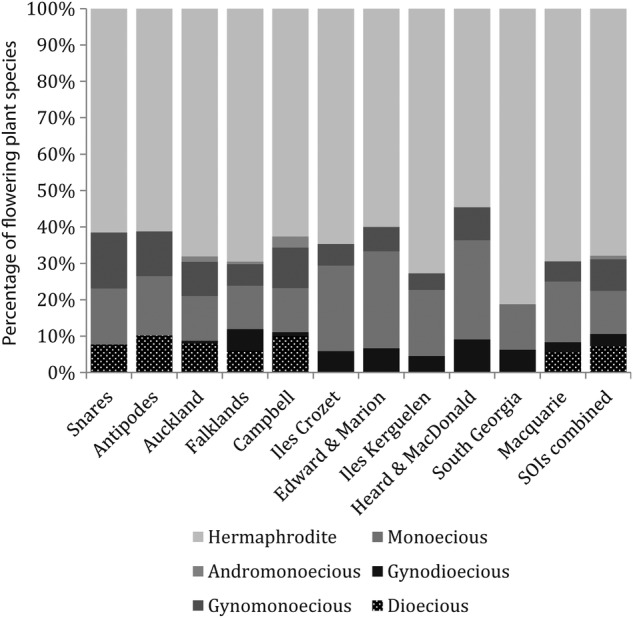
The frequency of breeding system classes among flowering plants native to 11 Southern Ocean Island groups. Arrangement of island groups along the horizontal axis follows Figure 1. See Table 1 for breeding system definitions.

Hermaphroditism was the most common breeding system overall (218 species, 67.9 %), followed by monoecy (38, 11.8 %), gynomonoecy (28, 8.7 %), dioecy (23, 7.1 %), gynodioecy (11, 3.4 %) and andromonoecy (3, 0.9 %). The number of species with hermaphroditic vs. other breeding systems did not differ significantly among the 11 island groups (*χ*^2^ = 5.31, *P* > 0.05, df = 10; Fig. 3). The proportion of species with gender dimorphic vs. gender monomorphic breeding systems did not differ significantly between the SOI flora and either the Stewart Island flora or the alpine flora of Patagonia (*χ*^2^ = 3.50, *P* > 0.05, df = 1; *χ*^2^ = 1.90, *P* > 0.05, df = 1 respectively). Dioecy only featured in the floras of the Falklands Islands and island groups in the south Pacific Province, where it occurred in up to 10.2 % of species (Table 2). The only gender dimorphic species found on other islands was *Acaena magellanica*.

Generalized linear models of species traits found that gender dimorphy was not associated with anemophily but was positively associated with fleshy fruit (Table 3). Genera shared with New Zealand or occurring in the Pacific province tended to have a greater proportion of gender dimorphic SOI species than other genera; however, gender dimorphism was negatively associated with the total number of biogeographic provinces occupied. Among islands, the number of species with dimorphic breeding systems was not explained by island latitude, province or climate zone.

## Discussion

Given the paucity of typical pollinating groups and the relentlessly cold and windy climatic conditions on SOIs, flowering plants could be expected to rely on wind pollination or self-fertilization for sexual reproduction (Smith 1984; Bergstrom *et al*. 1997; Schermann-Legionnet *et al*. 2007; Convey *et al*. 2010). However, the disadvantages of self-fertilization in small populations could favour the evolution of gender dimorphic breeding systems on SOIs as has been suggested for other island groups (Carlquist 1977; Bawa 1982; Sakai *et al*. 1995; Webb *et al*. 1999). This study has brought together the available data on SOI plants to test predictions concerning self-compatibility, anemophily and gender dimorphy. The SOI flora has a very high incidence of self-compatibility, 95.8 % of species for which information specifically from SOIs was available are described as at least partially self-compatible. Self-compatibility is thus considerably more common on SOIs than in New Zealand (63.9 %, Newstrom and Robertson 2005) or in the alpine flora of Patagonia (69.7 %, Arroyo and Squeo 1990), and also exceeds high values reported for other island groups, e.g. 80 % of native Galapagos Islands species (*N* = 55, Chamorro *et al*. 2012) and 85 % of native Juan Fernandez Islands species (*N* = 18, Bernardello *et al*. 2001). While information on a greater proportion of the SOI flora was obtained in this study compared with those of Chamorro *et al*. (2012) and Bernardello *et al*. (2001), the quality of the data is unknown and mostly stems from simple statements rather than experimentation, thus the level of self-compatibility might be overstated. However, even if further experimental work modifies this value, it still represents an extreme on a global scale.

Unlike self-compatibility, floral traits consistent with anemophily were no more common overall in the SOI region compared with southern New Zealand and southern South America. Anemophily did, however, show a relationship with climate zone; genera represented on subantarctic islands possessed a higher proportion of anemophilous species than genera restricted to cool temperate islands, and cool temperate island floras showed a higher incidence of species with floral traits suggestive of biotic pollination. The fact that no relationship was observed between anemophily and latitude reflects the influence of the Southern Ocean on the terrestrial flora of SOIs. The Antarctic Polar Frontal Zone, which is responsible for significantly cooler sea surface temperatures around subantarctic, as opposed to cool temperate, islands (Chown *et al*. 1998), varies in latitude from ∼48°S to 63°S depending on longitude and is remarkably stable apart from seasonal movements (Moore *et al*. 1999). Thus climates on the subantarctic islands of this study (south of the Polar Front) are considerably colder (∼4 °C mean drop in sea surface temperature, data from Chown *et al*. 1998) than cool temperate islands to the north of the front. This boundary also marks a dramatic drop in vascular plant species richness which is strongly linked to insect species richness (Chown *et al*. 1998).

Despite climatic constraints apparently favouring anemophily on harsher subantarctic islands, the fact that the largest class of floral types consisted of petaloid, often coloured, floral displays, indicates that biotic pollination cannot be ruled out as a means of sexual reproduction among SOI plants. Notes of insects observed visiting flowers of SOI species were found throughout the literature researched for this study; however, observations were seldom recorded systematically. It is highly likely that many plant species employ a mixed mating strategy combining opportunistic biotic pollination with the reproductive assurance of self-fertilization. For example, on Auckland and Campbell Islands, flower-visiting moths and flies are easily overlooked as they are only active during rare periods of sunshine, and nocturnal insects, including a native Orthopteran, are likely important pollinators (Lord *et al*. 2013; Lord unpubl. data). Such findings and studies of other island systems (e.g. Olesen and Valido 2003; Olesen *et al*. 2012) highlight the opportunity for novel plant–pollinator relationships to evolve on isolated islands. A further indication that biotic pollination is still important on SOIs is the finding that gender dimorphy was not associated with anemophily, so many gender dimorphic species must rely on biotic pollinators for sexual reproduction, as has been found by Lord *et al*. (2013) for two dioecious species on Campbell Island. However, the absence of dioecious species from all subantarctic islands apart from Macquarie Island suggests a reliance on cross-pollination might reduce the success of a species establishing and/or persisting on harsher subantarctic islands.

The frequency of gender dimorphy among SOI species was substantially higher than values reported for other high-latitude islands (e.g. Iceland and British Isles both 3 %; Baker and Cox 1984). While gender dimorphy at the species level was strongly related to fleshy fruits, suggesting advantages associated with long-distance dispersal (Bawa 1982; Sakai *et al*. 1995; Webb *et al*. 1999), at the genus-level gender dimorphy showed a strong effect of taxonomic affinity with the New Zealand flora, which has a high incidence of gender dimorphy (Webb *et al*. 1999). This supports the contention of Baker and Cox (1984) that a potential source flora with a high prevalence of dimorphy is a major factor in explaining patterns of breeding systems on islands. However, unlike the classic pacific examples of the evolution of gender dimorphy (Hawaiian Islands, Sakai *et al*. 1995; New Zealand, Webb *et al*. 1999), gender dimorphic breeding systems were not a feature of species-rich lineages on SOIs (which were generally lacking). Furthermore, no gynodioecious species were SOI endemics and all SOI endemic dioecious species were in genera with dioecious or dimorphic species elsewhere. So while a dimorphic breeding system is clearly a viable reproductive strategy in the Southern Ocean region, until more data are available on phylogenetic affinities among SOI plants and their mainland relatives, there is no clear evidence that gender dimorphic breeding systems have evolved *in situ* on these isolated islands.

## Conclusions

Very little experimental data exist concerning the reproductive ecology of SOI plants *in situ.* This lack of data is surprising given the relative simplicity of these floras and the long history of botanical study on many of these islands. Many SOI species show floral features consistent with biotic pollination and a number have been shown to be reliant on floral visitors for pollen transfer. Furthermore, species capable of autonomous self-fertilization can still benefit from out-crossing; for example, in a mainland study of gynodioecious *Fuchsia excorticata*, which occurs on Auckland Islands, fewer than 10 % of progeny derived from self-pollination in hermaphrodites survived and none had flowered after 11 years (Robertson *et al*. 2011). Virtually nothing is known about floral visitors to petaloid SOI species, but from the little information available it is clear that insect–plant interactions on SOIs require more research. Detailed studies such as those of Bergstrom *et al*. (1997), Schermann-Legionnet *et al*. (2007) and Lord *et al*. (2013) are required to definitively determine breeding systems and reliance on biotic pollinators, and the results of Lord *et al*. (2013) suggest that even nocturnal or flightless invertebrates may be capable of providing pollination services, so warrant closer study. Southern Ocean Island plants are being subjected to continued and increasing pressure from introduced species, human impacts and the likely effects of climate change (Meurk 1982; Smith and Steenkamp 1990; Copson and Whinam 2001; Chapuis *et al*. 2004; Le Roux *et al*. 2005; Shaw *et al*. 2005; McGlone *et al*. 2007; Convey *et al*. 2010). Management to assist recovery from disturbance and promote regeneration following restoration efforts requires an understanding of fundamental plant ecology including reproductive strategies.

## Sources of Funding

Funding for this project was provided by a University of Otago Research and Study Leave Grant and a Fulbright New Zealand Travel Award.

## Conflict of Interest Statement

None declared.

## Supporting Information

The following additional information is available in the online version of this article –

**Table S1.** List of all 321 flowering plant species used in analyses, their occurrence on 11 Southern Ocean Islands, breeding system, self-compatibility information (where available), floral traits and sources of information.

Additional Information
