# Supplementary material for: Patterns in floral traits and plant breeding systems on Southern Ocean Islands
Source: AoB Plants. 2015 Aug 18;7:plv095. doi: 10.1093/aobpla/plv095 (PMC4583772; doi:10.1093/aobpla/plv095)
Supplement: Additional Information [file supp_7_plv095_index.html]

Additional Information 

# Patterns in floral traits and plant breeding systems on Southern Ocean Islands

## Additional Information

Additional Information

- Additional Information - docx file
