# Supplementary material for: Patterns in floral traits and plant breeding systems on Southern Ocean Islands
Source: AoB Plants. 2015 Aug 18;7:plv095. doi: 10.1093/aobpla/plv095 (PMC4583772; doi:10.1093/aobpla/plv095)
Supplement: Additional Information [file supp_plv095_plv095supp_file.docx]

**Supplementary Information**

**Table 3**: Flowering plant species on Southern Ocean islands. I = Îles Crozet; P = Prince Edward & Marion Islands; S = The Snares; K = Îles Kerguelen; A = Antipodes; U = Auckland Islands; F = Falklands/Malvinas Islands; C = Campbell Island; H = Heard and MacDonald Islands; G = South Georgia; M = Macquarie Island; End = species restricted to the Southern Ocean region; FT=floral type: “a” = floral traits consistent with anemophily, “b” = showy flowers suggestive of biotic pollination, “ab” = floral traits not clearly consistent with either biotic pollination or anemophily, “minute” = very small solitary flowers; Comp = compatibility data for hermaphroditic species or morphs: SC = fully self-compatible, SCp = partially self-compatible, SI = self-incompatible, n.d. = no data; BSYS = breeding system: AM = andromonoecious, D = dioecious, GD = gynodioecious, GM = gynomonoecious, H = hermaphrodite, M = monoecious. Key to references: 1, Allan (1961); 2, Greene (1964); 3, Percival (1965); 4, Godley (1966); 5, Moore (1968); 6, Moore & Edgar (1970); 7, Lloyd (1972); 8, Raven & Raven (1976); 9, Walton (1979); 10, Walton (1982); 11, Moore (1983); 12, Godley (1985); 13, Webb et al. (1988); 14, Godley (1989); 15, Orchard (1989); 16, Arroyo & Squeo (1990); 17, Goldblatt et al. (1990); 18, Arroyo et al. (1992); 19, Australian Biological Resources Study (1993); 20, Martinez (1993); 21, Ibisch et al. (1996); 22, Bergstrom et al. (1997); 23, Edgar & Connor (2000); 24, Nicholls (2000); 25, Glenny (2004); 26, Broughton & MacAdam (2005); 27, Lehnebach et al. (2005); 28, Nilsen & Ågren (2006); 29, Clements et al. (2007); 30, Schermann-Legionett et al. (2007); 31, Wagstaff & Hennion (2007); 32, Bischoff (2008); 33, Heenan (2008); 34, Robertson et al. 2008; 35, Sciligo (2009); 36, Zavala-Gallo et al. (2010); 37, Webb et al. (1999); 38, Cerfonteyn et al. (2011); 39, Lord (2012); 40, Upson (2012); 41, de Lange et al. (2013); 42, Lord et al. (2013).

| **Family** | **Species** | **I** | **P** | **S** | **K** | **A** | **U** | **F** | **C** | **H** | **G** | **M** | **End** | **FT** | **Comp** | **BSYS** | **References** |
| --- | --- | --- | --- | --- | --- | --- | --- | --- | --- | --- | --- | --- | --- | --- | --- | --- | --- |
| **Eudicots** |  |  |  |  |  |  |  |  |  |  |  |  |  |  |  |  |  |
| Amaranthaceae | *Chenopodium macrospermum* Hook.f. | 0 | 0 | 0 | 0 | 0 | 0 | 1 | 0 | 0 | 0 | 0 | 0 | minute | SC | H | 5 |
|  | *Suaeda argentinensis* A.Soriano | 0 | 0 | 0 | 0 | 0 | 0 | 1 | 0 | 0 | 0 | 0 | 0 | minute | n.d. | GD | 5 |
| Apiaceae | *Anisotome acutifolia* (Kirk) Cockayne | 0 | 0 | 1 | 0 | 0 | 0 | 0 | 0 | 0 | 0 | 0 | 1 | b | - | D | 1 |
|  | *Anisotome antipoda* Hook.f. | 0 | 0 | 0 | 0 | 1 | 1 | 0 | 1 | 0 | 0 | 0 | 1 | b | - | D | 1, 14 |
|  | *Anisotome latifolia* Hook.f. | 0 | 0 | 0 | 0 | 0 | 1 | 0 | 1 | 0 | 0 | 0 | 1 | b | - | D | 1, 42 |
|  | *Apium australe* Thouars | 0 | 0 | 0 | 0 | 0 | 0 | 1 | 0 | 0 | 0 | 0 | 0 | b | n.d. | H | 5 |
|  | *Apium prostratum* subsp. *prostratum* var. *filiforme* (A.Rich.) Kirk | 0 | 0 | 0 | 0 | 1 | 0 | 0 | 0 | 0 | 0 | 0 | 0 | b | n.d. | H | 1 |
|  | *Azorella filamentosa* Lam. | 0 | 0 | 0 | 0 | 0 | 0 | 1 | 0 | 0 | 0 | 0 | 0 | ab | n.d. | GD | 5, 18 |
|  | *Azorella lycopodioides* Gaudich. | 0 | 0 | 0 | 0 | 0 | 0 | 1 | 0 | 0 | 0 | 0 | 0 | ab | SCp | H | 5, 16 |
|  | *Azorella macquariensis* Orchard | 0 | 0 | 0 | 0 | 0 | 0 | 0 | 0 | 0 | 0 | 1 | 1 | ab | n.d. | H | 15, 22 |
|  | *Azorella monantha* Clos | 0 | 0 | 0 | 0 | 0 | 0 | 1 | 0 | 0 | 0 | 0 | 0 | ab | n.d. | M / AM | (5?), 20 |
|  | *Azorella selago* Hook.f. | 1 | 1 | 0 | 1 | 0 | 0 | 1 | 0 | 1 | 0 | 1 | 0 | ab | n.d. | M | 5, 20, 38 |
|  | *Bolax gummifera* (Lam.) Spreng. | 0 | 0 | 0 | 0 | 0 | 0 | 1 | 0 | 0 | 0 | 0 | 0 | ab | - | D / GD | 5, 16 |
|  | *Hydrocotyle chamaemorus* Cham. & Schltdl. | 0 | 0 | 0 | 0 | 0 | 0 | 1 | 0 | 0 | 0 | 0 | 0 | ab | SC | H | 5 |
|  | *Hydrocotyle microphylla* A.Cunn. | 0 | 0 | 0 | 0 | 0 | 0 | 0 | 1 | 0 | 0 | 0 | 0 | ab | n.d. | H | 1 |
|  | *Hydrocotyle novae-zelandiae* DC | 0 | 0 | 0 | 0 | 0 | 0 | 0 | 0 | 0 | 0 | 1 | 0 | ab | n.d. | H | 1 |
|  | *Lilaeopsis macloviana* (Gand.) A.W.Hill | 0 | 0 | 0 | 0 | 0 | 0 | 1 | 0 | 0 | 0 | 0 | 0 | minute | n.d. | H | 5 |
|  | *Lilaeopsis novae-zelandiae* A.W.Hill | 0 | 0 | 0 | 0 | 0 | 1 | 0 | 0 | 0 | 0 | 0 | 0 | minute | n.d. | H | 1 |
|  | *Oreomyrrhis hookeri* Mathias & Constance | 0 | 0 | 0 | 0 | 0 | 0 | 1 | 0 | 0 | 0 | 0 | 0 | ab | n.d. | H | 5 |
|  | *Schizeilema ranunculus* Domin | 0 | 0 | 0 | 0 | 0 | 0 | 1 | 0 | 0 | 0 | 0 | 0 | ab | n.d. | H | 5 |
|  | *Schizeilema reniforme* Domin | 0 | 0 | 0 | 0 | 0 | 1 | 0 | 0 | 0 | 0 | 0 | 1 | ab | n.d. | H | 1 |
|  | *Stilbocarpa polaris* (Hombr. & Jacq.) A.Gray | 0 | 0 | 0 | 0 | 1 | 1 | 0 | 1 | 0 | 0 | 1 | 1 | b | n.d. | H | 1, 39 |
|  | *Stilbocarpa robusta* (Kirk) Cockayne | 0 | 0 | 1 | 0 | 0 | 0 | 0 | 0 | 0 | 0 | 0 | 0 | b | n.d. | H | 1 |
| Araliaceae | *Raukaua simplex* (G.Forst.) A.D.Mitch,Frodin & Heads | 0 | 0 | 0 | 0 | 0 | 1 | 0 | 0 | 0 | 0 | 0 | 0 | b | n.d. | M | 1 |
| Asteraceae | *Abrotanella emarginata* (Gaudich.) Cass. | 0 | 0 | 0 | 0 | 0 | 0 | 1 | 0 | 0 | 0 | 0 | 0 | ab | SCp | GM | 5 |
|  | *Abrotanella rosulata* (Hook.f.) Hook.f. | 0 | 0 | 0 | 0 | 0 | 1 | 0 | 1 | 0 | 0 | 0 | 1 | b | n.d. | GM | 1 |
|  | *Abrotanella spathulata* (Hook.f.) Hook.f. | 0 | 0 | 0 | 0 | 0 | 1 | 0 | 1 | 0 | 0 | 0 | 1 | b | n.d. | GM | 1 |
|  | *Agoseris coronopifolia* (d'Urv.) K.L.Chambers | 0 | 0 | 0 | 0 | 0 | 0 | 1 | 0 | 0 | 0 | 0 | 0 | b | SC | H | 5 |
|  | *Anaphalioides bellidioides* (G.Forst.) Glenny | 0 | 0 | 0 | 0 | 1 | 1 | 0 | 1 | 0 | 0 | 0 | 0 | b | SI | GM | 32 |
|  | *Baccharis magellanica* (Lam.) Pers. | 0 | 0 | 0 | 0 | 0 | 0 | 1 | 0 | 0 | 0 | 0 | 0 | ab | - | D | 5 |
|  | *Brachyglottis stewartiae* (J.B.Armstr.) B.Nord | 0 | 0 | 1 | 0 | 0 | 0 | 0 | 0 | 0 | 0 | 0 | 0 | b | n.d. | GM | 1 |
|  | *Brachyscome radicata* Hook.f. | 0 | 0 | 0 | 0 | 0 | 0 | 0 | 1 | 0 | 0 | 0 | 0 | b | n.d. | GM | 1 |
|  | *Cheveulia lycopodioides* (D’Urv.) DC. | 0 | 0 | 0 | 0 | 0 | 0 | 1 | 0 | 0 | 0 | 0 | 1 | ab | n.d. | GM | 5 |
|  | *Chiliotrichum diffusum* (G.Forst.) Kuntze | 0 | 0 | 0 | 0 | 0 | 0 | 1 | 0 | 0 | 0 | 0 | 0 | b | SI | H | 5 |
|  | *Craspedia uniflora* var. *subhispida* Allan | 0 | 0 | 0 | 0 | 0 | 0 | 0 | 1 | 0 | 0 | 0 | 1 | b | n.d. | H | 1 |
|  | *Damnemenia vernicosa* (Hook.f.) D.R.Given | 0 | 0 | 0 | 0 | 0 | 1 | 0 | 1 | 0 | 0 | 0 | 1 | b | SC | GM | 42 |
|  | *Erigeron incertus* (d'Urv.) Skottsb. | 0 | 0 | 0 | 0 | 0 | 0 | 1 | 0 | 0 | 0 | 0 | 1 | b | n.d. | GM | 5, 40 |
|  | *Euchiton involucratus* (G.Forst.) Holub. | 0 | 0 | 0 | 0 | 0 | 1 | 0 | 0 | 0 | 0 | 0 | 0 | b | n.d. | GM | 13 |
|  | *Gamochaeta americana* (Mill.) Weddell | 0 | 0 | 0 | 0 | 0 | 0 | 1 | 0 | 0 | 0 | 0 | 1 | a | n.d. | GM | 5 |
|  | *Gamochaeta antarctica* (Hook.f.) Cabrera | 0 | 0 | 0 | 0 | 0 | 0 | 1 | 0 | 0 | 0 | 0 | 1 | a | SC | H | 5, 40 |
|  | *Gamochaeta malvinensis* (H.Koyama) T.F.Dudley | 0 | 0 | 0 | 0 | 0 | 0 | 1 | 0 | 0 | 0 | 0 | 1 | a | SC | H | 5, 40 |
|  | *Gamochaeta spiciformis* (Sch.Bip.) Cabrera | 0 | 0 | 0 | 0 | 0 | 0 | 1 | 0 | 0 | 0 | 0 | 0 | a | SCp | H | (5?), 16 |
|  | *Hieracium antarcticum* d'Urv. | 0 | 0 | 0 | 0 | 0 | 0 | 1 | 0 | 0 | 0 | 0 | 0 | b | SCp | H | 5 |
|  | *Hieracium patagonicum* Hook.f. | 0 | 0 | 0 | 0 | 0 | 0 | 1 | 0 | 0 | 0 | 0 | 0 | b | SC | H | 5 |
|  | *Hypochaeris arenaria* Gaudich. | 0 | 0 | 0 | 0 | 0 | 0 | 1 | 0 | 0 | 0 | 0 | 0 | b | n.d. | H | 5, 16 |
|  | *Lagenophora nudicaulis* (Comm. Ex Lam.) Dusén | 0 | 0 | 0 | 0 | 0 | 0 | 1 | 0 | 0 | 0 | 0 | 0 | b | n.d. | GM | 5 |
|  | *Lagenophora petiolata* Hook.f. | 0 | 0 | 0 | 0 | 1 | 1 | 0 | 1 | 0 | 0 | 0 | 0 | b | n.d. | GM | 1 |
|  | *Leptinella dispersa* (D.G.Lloyd) D.G.Lloyd & C.J.Webb | 0 | 0 | 0 | 0 | 0 | 0 | 0 | 1 | 0 | 0 | 0 | 0 | b | - | D / M | 7 |
|  | *Leptinella lanata* Hook.f. | 0 | 0 | 0 | 0 | 0 | 1 | 0 | 1 | 0 | 0 | 0 | 1 | b | n.d. | M | 7 |
|  | *Leptinella plumosa* Hook.f. | 1 | 1 | 0 | 1 | 1 | 1 | 0 | 1 | 1 | 0 | 1 | 1 | b | n.d. | M | 7 |
|  | *Leptinella potentillina* F.Muell. | 0 | 0 | 0 | 0 | 0 | 1 | 0 | 0 | 0 | 0 | 0 | 0 | b | n.d. | M | 7 |
|  | *Leptinella scariosa* Cass. | 0 | 0 | 0 | 0 | 0 | 0 | 1 | 0 | 0 | 0 | 0 | 0 | b | - | D | 5, 7 |
|  | *Leucheria suaveolens* (d'Urv) Speg. | 0 | 0 | 0 | 0 | 0 | 0 | 1 | 0 | 0 | 0 | 0 | 1 | b | n.d. | H | 5, 40 |
|  | *Nassauvia gaudichaudii* (Cass.) Cass. Ex Gaudich. | 0 | 0 | 0 | 0 | 0 | 0 | 1 | 0 | 0 | 0 | 0 | 1 | b | n.d. | H | 5 |
|  | *Nassauvia serpens* d'Urv. | 0 | 0 | 0 | 0 | 0 | 0 | 1 | 0 | 0 | 0 | 0 | 1 | b | n.d. | H | 5, 40 |
|  | *Olearia lyallii* Hook.f. | 0 | 0 | 1 | 0 | 0 | 0 | 0 | 0 | 0 | 0 | 0 | 1 | b | n.d. | GM | 1 |
|  | *Ozothamnus leptophyllus* (G.Forst.) Breitw. & J.M. Ward | 0 | 0 | 0 | 0 | 0 | 1 | 0 | 0 | 0 | 0 | 0 | 0 | b | n.d. | H | 1 |
|  | *Perezia recurvata* Less. | 0 | 0 | 0 | 0 | 0 | 0 | 1 | 0 | 0 | 0 | 0 | 0 | b | SI | H | 5 |
|  | *Pleurophyllum criniferum* Hook.f. | 0 | 0 | 0 | 0 | 1 | 1 | 0 | 1 | 0 | 0 | 0 | 1 | b | SC | GM | 24, 42 |
|  | *Pleurophyllum hookeri* Buchanan | 0 | 0 | 0 | 0 | 0 | 1 | 0 | 1 | 0 | 0 | 1 | 1 | b | SI | GM | 42 |
|  | *Pleurophyllum speciosum* Hook.f. | 0 | 0 | 0 | 0 | 0 | 1 | 0 | 1 | 0 | 0 | 0 | 1 | b | SI | GM | 24 |
|  | *Pseudognaphalium luteoalbum* (L.) Hilliard et B.L.Burtt | 0 | 0 | 0 | 0 | 1 | 0 | 0 | 0 | 0 | 0 | 0 | 0 | b | n.d. | GM | 1 |
|  | *Senecio biserratus* Belcher | 0 | 0 | 0 | 0 | 0 | 1 | 0 | 0 | 0 | 0 | 0 | 1 | b | n.d. | GM | 1 |
|  | *Senecio candidans* DC. | 0 | 0 | 0 | 0 | 0 | 0 | 1 | 0 | 0 | 0 | 0 | 0 | b | n.d. | H | 5 |
|  | *Senecio littoralis* Gaudich. | 0 | 0 | 0 | 0 | 0 | 0 | 1 | 0 | 0 | 0 | 0 | 1 | b | n.d. | GM | 5 |
|  | *Senecio radiolatus* subsp. *antipodus* (Kirk) C.J.Webb | 0 | 0 | 0 | 0 | 1 | 0 | 0 | 0 | 0 | 0 | 0 | 1 | b | n.d. | H | 14 |
|  | *Senecio vaginatus* Hook. & Arn. | 0 | 0 | 0 | 0 | 0 | 0 | 1 | 0 | 0 | 0 | 0 | 1 | b | n.d. | GM | 5 |
|  | *Symphyotrichum vahlii* (Gaudich.) G.L.Nesom | 0 | 0 | 0 | 0 | 0 | 0 | 1 | 0 | 0 | 0 | 0 | 0 | b | n.d. | GM | 5 |
|  | *Taraxacum gilliesii* Hook. & Arn. | 0 | 0 | 0 | 0 | 0 | 0 | 1 | 0 | 0 | 0 | 0 | 0 | b | n.d. | H | 5 |
|  | *Taraxacum magellanicum* Comm. Ex Sch.Bip. | 0 | 0 | 0 | 0 | 1 | 1 | 0 | 1 | 0 | 0 | 0 | 0 | b | n.d. | H | 1 |
| Boraginaceae | *Myosotis antarctica* Hook.f. | 0 | 0 | 0 | 0 | 0 | 0 | 0 | 1 | 0 | 0 | 0 | 1 | b | n.d. | H | 1 |
|  | *Myosotis capitata* Hook.f. | 0 | 0 | 0 | 0 | 0 | 1 | 0 | 1 | 0 | 0 | 0 | 1 | b | n.d. | H | 1 |
| Brassicaceae | *Cardamine corymbosa* Hook.f. | 0 | 0 | 0 | 0 | 1 | 1 | 0 | 1 | 0 | 0 | 1 | 1 | b | SC | H | 14, 22 |
|  | *Cardamine depressa* Hook.f. | 0 | 0 | 0 | 0 | 0 | 1 | 0 | 1 | 0 | 0 | 0 | 0 | b | n.d. | H | 1 |
|  | *Cardamine glacialis* (G.Forst.) DC. | 0 | 0 | 0 | 0 | 0 | 0 | 1 | 0 | 0 | 0 | 0 | 0 | b | SC | H | 5 |
|  | *Cardamine latior* Heenan | 0 | 0 | 0 | 0 | 0 | 1 | 0 | 0 | 0 | 0 | 0 | 1 | b | n.d. | H | 33 |
|  | *Cardamine subcarnosa* (Hook.f.) Allan | 0 | 0 | 0 | 0 | 0 | 0 | 0 | 1 | 0 | 0 | 0 | 1 | b | n.d. | H | 1 |
|  | *Draba funiculosa* Hook.f. | 0 | 0 | 0 | 0 | 0 | 0 | 1 | 0 | 0 | 0 | 0 | 0 | b | SC | H | 5 |
|  | *Draba magellanica* Lam. | 0 | 0 | 0 | 0 | 0 | 0 | 1 | 0 | 0 | 0 | 0 | 0 | b | SC | H | 5 |
|  | *Coronopus didymus* (L.) Sm. | 0 | 0 | 0 | 0 | 0 | 0 | 1 | 0 | 0 | 0 | 0 | 0 | b | n.d. | H | 5 |
|  | *Lepidium limenophylax* de Lange, B.D.Rance et D.A.Norton | 0 | 0 | 1 | 0 | 0 | 1 | 0 | 0 | 0 | 0 | 0 | 1 | b | n.d. | H | 41 |
|  | *Lepidium oligodontum* de Lange et Heenan | 0 | 0 | 0 | 0 | 1 | 0 | 0 | 0 | 0 | 0 | 0 | 1 | b | n.d. | H | 41 |
|  | *Phlebolobium maclovianum* (d'Urv.) O.E.Schulz | 0 | 0 | 0 | 0 | 0 | 0 | 1 | 0 | 0 | 0 | 0 | 1 | b | n.d. | H | 5 |
|  | *Pringlea antiscorbutica* R.Br.ex Hook.f. | 1 | 1 | 0 | 1 | 0 | 0 | 0 | 0 | 1 | 0 | 0 | 1 | b | SC | H | 30 |
| Calceolariaceae | *Calceolaria biflora* Lam. | 0 | 0 | 0 | 0 | 0 | 0 | 1 | 0 | 0 | 0 | 0 | 0 | b | SC | H | 5 |
|  | *Calceolaria fothergillii* Aiton | 0 | 0 | 0 | 0 | 0 | 0 | 1 | 0 | 0 | 0 | 0 | 1 | b | n.d. | H | 5 |
| Callitrichaceae | *Callitriche antarctica* Engelm. Ex Hegelm. | 1 | 1 | 1 | 1 | 1 | 1 | 1 | 1 | 1 | 1 | 1 | 0 | minute | n.d. | H | 2, 5, 10 |
|  | *Callitriche aucklandica* R.Mason | 0 | 0 | 0 | 0 | 0 | 1 | 0 | 0 | 0 | 0 | 0 | 1 | minute | n.d. | H | 1 |
| Calyceraceae | *Nastanthus falklandicus* D.M.Moore | 0 | 0 | 0 | 0 | 0 | 0 | 1 | 0 | 0 | 0 | 0 | 1 | ab | n.d. | H | 5, 36 |
| Caprifoliaceae | *Valeriana sedifolia* d'Urv. | 0 | 0 | 0 | 0 | 0 | 0 | 1 | 0 | 0 | 0 | 0 | 0 | b | - | D | 5 |
| Caryophyllaceae | *Cerastium arvense* L. | 0 | 0 | 0 | 0 | 0 | 0 | 1 | 0 | 0 | 0 | 0 | 0 | b | SC | H | 5 |
|  | *Colobanthus affinis* (Hook.) Hook.f. | 0 | 0 | 0 | 0 | 0 | 0 | 0 | 0 | 0 | 0 | 1 | 0 | ab | SCp | H | 1, 18 |
|  | *Colobanthus apetalus* (Labill.) Druce | 0 | 0 | 0 | 0 | 1 | 1 | 0 | 1 | 0 | 0 | 1 | 0 | ab | n.d. | H | 1 |
|  | *Colobanthus hookeri* Cheeseman | 0 | 0 | 0 | 0 | 0 | 1 | 0 | 1 | 0 | 0 | 0 | 1 | ab | n.d. | H | 1 |
|  | *Colobanthus kerguelensis* Hook.f. | 1 | 1 | 0 | 1 | 0 | 0 | 0 | 0 | 1 | 0 | 0 | 1 | ab | n.d. | H | 19 |
|  | *Colobanthus muscoides* Hook.f. | 0 | 0 | 1 | 0 | 1 | 1 | 0 | 1 | 0 | 0 | 1 | 1 | ab | n.d. | H | 1 |
|  | *Colobanthus quitensis* (Kunth) Bartl. | 0 | 0 | 0 | 0 | 0 | 0 | 1 | 0 | 0 | 1 | 0 | 0 | ab | SC | H | 2, 5, 10 |
|  | *Colobanthus subulatus* (d'Urv.) Hook.f. | 0 | 0 | 0 | 0 | 0 | 0 | 1 | 0 | 0 | 1 | 0 | 0 | ab | SC | H | 2, 5, 10 |
|  | *Spergularia marina* (L.) Besser | 0 | 0 | 0 | 0 | 0 | 0 | 1 | 0 | 0 | 0 | 0 | 0 | b | SC | H | 5 |
|  | *Stellaria debilis* d'Urv. | 0 | 0 | 0 | 0 | 0 | 0 | 1 | 0 | 0 | 0 | 0 | 0 | ab | SC | H | 5, 11, 16 |
|  | *Stellaria decipiens* Hook.f. | 0 | 0 | 0 | 0 | 1 | 1 | 0 | 1 | 0 | 0 | 0 | 1 | b | n.d. | H | 13 |
|  | *Stellaria parviflora* Banks & Sol. Ex Hook.f. | 0 | 0 | 0 | 0 | 0 | 0 | 0 | 0 | 0 | 0 | 1 | 0 | b | n.d. | H | 13 |
| Crassulaceae | *Crassula moschata* G.Forst. | 1 | 1 | 1 | 1 | 1 | 1 | 1 | 1 | 0 | 0 | 1 | 0 | minute | n.d. | H | 5 |
| Droseraceae | *Drosera stenopetala* Hook.f. | 0 | 0 | 0 | 0 | 0 | 1 | 0 | 1 | 0 | 0 | 0 | 0 | b | SC | H | 1, 35 |
|  | *Drosera uniflora* Willd. | 0 | 0 | 0 | 0 | 0 | 0 | 1 | 0 | 0 | 0 | 0 | 0 | b | n.d. | H | 5 |
| Ericaceae | *Cyathodes empetrifolia* (Hook.f.) Hook.f. | 0 | 0 | 0 | 0 | 0 | 1 | 0 | 1 | 0 | 0 | 0 | 0 | b | n.d. | H | 1 |
|  | *Dracophyllum longifolium* (J.R.Forst. & G.Forst) R.Br. Ex Roem. & Schult. | 0 | 0 | 0 | 0 | 0 | 1 | 0 | 1 | 0 | 0 | 0 | 0 | b | n.d. | H | 1 |
|  | *Dracophyllum scoparium* Hook.f. | 0 | 0 | 0 | 0 | 0 | 0 | 0 | 1 | 0 | 0 | 0 | 0 | b | n.d. | H | 1 |
|  | *Empetrum rubrum* Vahl ex Willd. | 0 | 0 | 0 | 0 | 0 | 0 | 1 | 0 | 0 | 0 | 0 | 0 | b | - | D | 5 |
|  | *Gaultheria antarctica* Hook.f. | 0 | 0 | 0 | 0 | 0 | 0 | 1 | 0 | 0 | 0 | 0 | 0 | b | - | D | 5 |
|  | *Gaultheria pumila* (L.f.) D.J.Middleton | 0 | 0 | 0 | 0 | 0 | 0 | 1 | 0 | 0 | 0 | 0 | 0 | b | - | D | 4, 5 |
| Gentianaceae | *Gentianella antarctica* (Kirk) T.N.Ho & S.W.Liu | 0 | 0 | 0 | 0 | 0 | 0 | 1 | 1 | 0 | 0 | 0 | 0 | b | SC | GD / H | 11, 14 |
|  | *Gentianella antipoda* (Kirk) T.N.Ho & S.W.Liu | 0 | 0 | 0 | 0 | 1 | 0 | 0 | 0 | 0 | 0 | 0 | 1 | b | SC | GM | 14 |
|  | *Gentianella cerina* (Hook.f.) T.N.Ho & S.W.Liu | 0 | 0 | 0 | 0 | 0 | 1 | 0 | 0 | 0 | 0 | 0 | 1 | b | n.d. | H | 25 |
|  | *Gentianella concinnna* (Hook.f.) T.N.Ho & S.W.Liu | 0 | 0 | 0 | 0 | 0 | 1 | 0 | 0 | 0 | 0 | 0 | 1 | b | n.d. | H | 25 |
|  | *Gentianella magellanica* (Gaudich.) Fabris | 0 | 0 | 0 | 0 | 0 | 0 | 1 | 0 | 0 | 0 | 0 | 1 | b | SC | H | 5 |
| Geraniaceae | *Geranium microphyllum* Hook.f. | 0 | 0 | 0 | 0 | 1 | 1 | 0 | 1 | 0 | 0 | 0 | 0 | b | n.d. | H | 1 |
| Gunneraceae | *Gunnera magellanica* Lam. | 0 | 0 | 0 | 0 | 0 | 0 | 1 | 0 | 0 | 0 | 0 | 0 | ab | - | D | 5 |
| Haloragaceae | *Gonocarpus aggregatus* (Buchanan) Orchard | 0 | 0 | 0 | 0 | 0 | 1 | 0 | 1 | 0 | 0 | 0 | 0 | minute | n.d. | H / M | 1 |
| Lamiaceae | *Scutellaria nummulariifolia* Hook.f. | 0 | 0 | 0 | 0 | 0 | 0 | 1 | 0 | 0 | 0 | 0 | 0 | b | n.d. | H | 5 |
| Lobeliaceae | *Lobelia arenaria* (Hook.f.)Heenan & de Lange | 0 | 0 | 0 | 0 | 1 | 1 | 0 | 1 | 0 | 0 | 0 | 0 | b | n.d. | H | 1 |
|  | *Lobelia pratiana* Gaudich. Ex Lammers | 0 | 0 | 0 | 0 | 0 | 0 | 1 | 0 | 0 | 0 | 0 | 0 | b | SC | H | 5 |
| Myrsinaceae | *Myrsine divaricata* A.Cunn. | 0 | 0 | 0 | 0 | 0 | 1 | 0 | 1 | 0 | 0 | 0 | 0 | b | - | D | 37 |
| Myrtaceae | *Metrosideros umbellata* Cav. | 0 | 0 | 0 | 0 | 0 | 1 | 0 | 0 | 0 | 0 | 0 | 0 | b | n.d. | H | 1 |
|  | *Myrteola nummularia* (Lam.) O.Berg | 0 | 0 | 0 | 0 | 0 | 0 | 1 | 0 | 0 | 0 | 0 | 0 | b | SC | H | 5 |
| Onagraceae | *Epilobium alsinoides* A.Cunn. | 0 | 0 | 0 | 0 | 1 | 1 | 0 | 1 | 0 | 0 | 0 | 0 | b | SC | H | 8, 14 |
|  | *Epilobium brunnescens* (Cockayne) P.H.Raven & Engelhorn | 0 | 0 | 0 | 0 | 0 | 1 | 0 | 1 | 0 | 0 | 1 | 0 | b | SC | H | 8 |
|  | *Epilobium ciliatum* Raf. | 0 | 0 | 0 | 0 | 0 | 0 | 1 | 0 | 0 | 0 | 0 | 0 | b | n.d. | H | 26 |
|  | *Epilobium confertifolium* Hook.f. | 0 | 0 | 0 | 0 | 0 | 1 | 0 | 1 | 0 | 0 | 0 | 1 | b | SC | H | 8 |
|  | *Epilobium pedunculare* A.Cunn. | 0 | 0 | 0 | 0 | 1 | 1 | 0 | 1 | 0 | 0 | 1 | 0 | b | SC | H | 8, 14, 22 |
|  | *Epilobium pernitens* Cockayne & Allan | 0 | 0 | 0 | 0 | 0 | 0 | 0 | 1 | 0 | 0 | 0 | 0 | b | SC | H | 8 |
|  | *Fuchsia excorticata* (G.Forst.) L.f. | 0 | 0 | 0 | 0 | 0 | 1 | 0 | 0 | 0 | 0 | 0 | 0 | b | SC | GD | 12, 34 |
| Orobanchaceae | *Euphrasia antarctica* Benth. | 0 | 0 | 0 | 0 | 0 | 0 | 1 | 0 | 0 | 0 | 0 | 0 | b | n.d. | H | 5 |
| Oxalidaceae | *Oxalis enneaphylla* Cav. | 0 | 0 | 0 | 0 | 0 | 0 | 1 | 0 | 0 | 0 | 0 | 0 | b | SI | H | 5 |
| Plantaginaceae | *Plantago aucklandica* Hook.f. | 0 | 0 | 0 | 0 | 0 | 1 | 0 | 0 | 0 | 0 | 0 | 1 | a | n.d. | H | 1 |
|  | *Plantago barbata* G.Forst. | 0 | 0 | 0 | 0 | 0 | 0 | 1 | 0 | 0 | 0 | 0 | 0 | a | SC | H | 5 |
|  | *Plantago maritima* L. | 0 | 0 | 0 | 0 | 0 | 0 | 1 | 0 | 0 | 0 | 0 | 0 | a | SC | GD | 5, 28 |
|  | *Plantago moorei* Rahn. | 0 | 0 | 0 | 0 | 0 | 0 | 1 | 0 | 0 | 0 | 0 | 1 | a | n.d. | H | 26, 40 |
|  | *Plantago triantha* Spreng. | 0 | 0 | 0 | 0 | 0 | 1 | 0 | 0 | 0 | 0 | 0 | 1 | a | n.d. | H | 1 |
|  | *Veronica benthamii* Hook.f. | 0 | 0 | 0 | 0 | 0 | 1 | 0 | 1 | 0 | 0 | 0 | 1 | b | SC | H | 42 |
|  | *Veronica elliptica* G.Forst. | 0 | 0 | 1 | 0 | 0 | 1 | 1 | 1 | 0 | 0 | 0 | 0 | b | n.d. | H | 5 |
|  | *Veronica odora* Hook.f. | 0 | 0 | 0 | 0 | 0 | 1 | 0 | 0 | 0 | 0 | 0 | 0 | b | n.d. | H | 1 |
|  | *Veronica salicifolia* G.Forst. | 0 | 0 | 0 | 0 | 0 | 1 | 0 | 0 | 0 | 0 | 0 | 0 | b | n.d. | H | 1 |
| Plumbaginaceae | *Armeria maritima* (Mill.) Willd. | 0 | 0 | 0 | 0 | 0 | 0 | 1 | 0 | 0 | 0 | 0 | 0 | b | SC | H | 5 |
| Polygonaceae | *Polygonum maritimum* L. | 0 | 0 | 0 | 0 | 0 | 0 | 1 | 0 | 0 | 0 | 0 | 0 | b | n.d. | H | 5 |
|  | *Rumex flexuosus* Sol. Ex G.Forst. | 0 | 0 | 0 | 0 | 0 | 0 | 0 | 1 | 0 | 0 | 0 | 0 | a | n.d. | H | 1 |
|  | *Rumex magellanicus* Campd. | 0 | 0 | 0 | 0 | 0 | 0 | 1 | 0 | 0 | 0 | 0 | 0 | a | n.d. | H | 5, 40 |
|  | *Rumex neglectus* Kirk | 0 | 0 | 0 | 0 | 1 | 1 | 0 | 0 | 0 | 0 | 0 | 0 | a | n.d. | H | 1 |
| Portulacaceae | *Lyallia kerguelensis* Hook.f. | 0 | 0 | 0 | 1 | 0 | 0 | 0 | 0 | 0 | 0 | 0 | 1 | ab | n.d. | H | 31 |
|  | *Montia australasica* (Hook.f.) Pax & K.Hoffm. | 0 | 0 | 0 | 0 | 0 | 1 | 0 | 1 | 0 | 0 | 0 | 0 | b | n.d. | H | 1 |
|  | *Montia fontana* L. | 1 | 1 | 0 | 1 | 1 | 1 | 1 | 1 | 1 | 1 | 1 | 0 | minute | SC | H | 2, 5, 10, 22 |
| Primulaceae | *Anagallis alternifolia* var. *densifolia* Hook.f. | 0 | 0 | 0 | 0 | 0 | 0 | 1 | 0 | 0 | 0 | 0 | 0 | b | SC | H | 5 |
|  | *Primula magellanica* Lehm. | 0 | 0 | 0 | 0 | 0 | 0 | 1 | 0 | 0 | 0 | 0 | 0 | b | SC | H | 5 |
|  | *Samolus repens* Pers. | 0 | 0 | 0 | 0 | 0 | 1 | 1 | 0 | 0 | 0 | 0 | 0 | b | n.d. | H | 1, 40 |
| Ranunculaceae | *Hamadryas argentea* Hook.f. | 0 | 0 | 0 | 0 | 0 | 0 | 1 | 0 | 0 | 0 | 0 | 1 | b | - | D | 5 |
|  | *Psychrophila appendiculata* Bercht. & J.Presl | 0 | 0 | 0 | 0 | 0 | 0 | 1 | 0 | 0 | 0 | 0 | 0 | b | SC | H | 5 |
|  | *Psychrophila sagittata* (Cav.) Bercht. & J.Presl | 0 | 0 | 0 | 0 | 0 | 0 | 1 | 0 | 0 | 0 | 0 | 0 | b | SC | H | 5 |
|  | *Ranunculus acaulis* DC. | 0 | 0 | 0 | 0 | 0 | 1 | 1 | 0 | 0 | 0 | 0 | 0 | b | n.d. | H | 5 |
|  | *Ranunculus biternatus* Sm. | 1 | 1 | 0 | 1 | 0 | 0 | 1 | 0 | 0 | 1 | 0 | 0 | b | SC | H | 2, 5, 10 |
|  | *Ranunculus crassipes* Hook.f. | 0 | 0 | 0 | 0 | 0 | 0 | 0 | 0 | 1 | 0 | 1 | 1 | b | n.d. | H | 19 |
|  | *Ranunculus hydrophilus* Gaudich. Ex Mirb. | 0 | 0 | 0 | 0 | 0 | 0 | 1 | 0 | 0 | 0 | 0 | 0 | b | n.d. | H | 5 |
|  | *Ranunculus maclovianus* d'Urville | 0 | 0 | 0 | 0 | 0 | 0 | 1 | 0 | 0 | 0 | 0 | 0 | b | SC | H | 5 |
|  | *Ranunculus moseleyi* Hook.f. | 0 | 1 | 0 | 1 | 0 | 0 | 0 | 0 | 0 | 0 | 0 | 1 | b | n.d. | H | 3 |
|  | *Ranunculus pinguis* Hook.f. | 0 | 0 | 0 | 0 | 0 | 1 | 0 | 1 | 0 | 0 | 0 | 1 | b | n.d. | H | 13 |
|  | *Ranunculus pseudotrullifolius* Skottsb. | 0 | 0 | 0 | 1 | 0 | 0 | 1 | 0 | 0 | 0 | 0 | 1 | b | n.d. | H | 5 |
|  | *Ranunculus sericocephalus* Hook.f. | 0 | 0 | 0 | 0 | 0 | 0 | 1 | 0 | 0 | 0 | 0 | 0 | b | SC | H | 5 |
|  | *Ranunculus subscaposus* Hook.f. | 0 | 0 | 0 | 0 | 0 | 1 | 0 | 1 | 0 | 0 | 0 | 1 | b | n.d. | H | 13 |
|  | *Ranunculus trullifolius* Hook.f. | 0 | 0 | 0 | 0 | 0 | 0 | 1 | 0 | 0 | 0 | 0 | 0 | b | n.d. | H | 5 |
| Rosaceae | *Acaena anserinifolia* (J.R.Forst. & G.Forst) J.B. Armstr. | 0 | 0 | 0 | 0 | 0 | 1 | 0 | 1 | 0 | 0 | 0 | 0 | ab | n.d. | H | 13 |
|  | *Acaena antarctica* Hook.f. | 0 | 0 | 0 | 0 | 0 | 0 | 1 | 0 | 0 | 0 | 0 | 0 | ab | SC | H | 5, 16 |
|  | *Acaena lucida* (Aiton) Vahl | 0 | 0 | 0 | 0 | 0 | 0 | 1 | 0 | 0 | 0 | 0 | 0 | ab | SC | H | 5 |
|  | *Acaena magellanica* (Lam.) Vahl | 1 | 1 | 0 | 1 | 0 | 0 | 1 | 0 | 1 | 1 | 1 | 0 | ab | SC | GD | 2, 5, 9, 10 |
|  | Acaena minor (Hook.f.) Allan | 0 | 0 | 0 | 0 | 1 | 1 | 0 | 1 | 0 | 0 | 1 | 1 | ab | n.d. | H | 13 |
|  | *Acaena novae-zelandiae* Kirk | 0 | 0 | 0 | 0 | 0 | 0 | 0 | 1 | 0 | 0 | 0 | 0 | ab | n.d. | H | 13 |
|  | *Acaena ovalifolia* Ruiz & Pav. | 0 | 0 | 0 | 0 | 0 | 0 | 1 | 0 | 0 | 0 | 0 | 0 | ab | SC | H | 5 |
|  | *Acaena pumila* Vahl | 0 | 0 | 0 | 0 | 0 | 0 | 1 | 0 | 0 | 0 | 0 | 0 | ab | n.d. | H | 5 |
|  | *Acaena tenera* Albov. | 0 | 0 | 0 | 0 | 0 | 0 | 0 | 0 | 0 | 1 | 0 | 1 | ab | SC | H | 10 |
|  | *Geum parviflorum* Hook.f. | 0 | 0 | 0 | 0 | 0 | 1 | 0 | 0 | 0 | 0 | 0 | 0 | b | n.d. | H | 1 |
|  | *Rubus geoides* Sm. | 0 | 0 | 0 | 0 | 0 | 0 | 1 | 0 | 0 | 0 | 0 | 0 | b | SC | H | 5 |
| Rubiaceae | *Coprosma ciliata* Hook.f. | 0 | 0 | 0 | 0 | 1 | 1 | 0 | 1 | 0 | 0 | 0 | 0 | a | - | D | 1 |
|  | *Coprosma cuneata* Hook.f. | 0 | 0 | 0 | 0 | 0 | 1 | 0 | 1 | 0 | 0 | 0 | 0 | a | - | D | 1 |
|  | *Coprosma foetidissima* J.R.Forst. & G.Forst. | 0 | 0 | 0 | 0 | 0 | 1 | 0 | 0 | 0 | 0 | 0 | 0 | a | - | D | 1 |
|  | *Coprosma perpusilla* Colenso | 0 | 0 | 0 | 0 | 1 | 1 | 0 | 1 | 0 | 0 | 1 | 0 | a | - | D | 1 |
|  | *Coprosma rugosa* Cheeseman | 0 | 0 | 0 | 0 | 1 | 0 | 0 | 0 | 0 | 0 | 0 | 0 | a | - | D | 1 |
|  | *Galium antarcticum* Hook.f. | 1 | 0 | 0 | 1 | 0 | 0 | 1 | 0 | 0 | 1 | 1 | 0 | ab | n.d. | H | 2, 5, 10, 11 |
|  | *Galium propinquum* A.Cunn. | 0 | 0 | 0 | 0 | 0 | 1 | 0 | 0 | 0 | 0 | 0 | 0 | ab | n.d. | H | 1 |
|  | *Nertera granadensis* (Mutis ex L.f.) Druce | 0 | 0 | 0 | 0 | 0 | 1 | 1 | 0 | 0 | 0 | 0 | 0 | a | n.d. | H | 5 |
| Santalaceae | *Nanodea muscosa* Banks ex C.F.Gaertn. | 0 | 0 | 0 | 0 | 0 | 0 | 1 | 0 | 0 | 0 | 0 | 1 | b | n.d. | H | 5 |
| Saxifragaceae | *Saxifraga magellanica* Poir. | 0 | 0 | 0 | 0 | 0 | 0 | 1 | 0 | 0 | 0 | 0 | 0 | b | SC | H | 5, 16 |
| Scrophulariaceae | *Limosella australis* R.Br. | 1 | 1 | 0 | 1 | 0 | 1 | 1 | 0 | 0 | 0 | 0 | 0 | minute | n.d. | H | 1, 5, 16 |
| Stylidiaceae | *Phyllachne clavigera* F.Muell. | 0 | 0 | 0 | 0 | 0 | 1 | 0 | 0 | 0 | 0 | 0 | 0 | b | n.d. | M | 1 |
|  | *Phyllachne colensoi* Berggr. | 0 | 0 | 0 | 0 | 0 | 0 | 0 | 1 | 0 | 0 | 0 | 0 | b | SC | M | 32 |
| Thymelaeaceae | *Drapetes lyallii* Hook.f. | 0 | 0 | 0 | 0 | 0 | 1 | 0 | 0 | 0 | 0 | 0 | 0 | b | n.d. | H | 1 |
|  | *Drapetes muscosus* Lam. | 0 | 0 | 0 | 0 | 0 | 0 | 1 | 0 | 0 | 0 | 0 | 0 | ab | n.d. | H | 5 |
| Urticaeae | *Urtica aucklandica* Hook.f. | 0 | 0 | 0 | 0 | 0 | 1 | 0 | 0 | 0 | 0 | 0 | 1 | ab | n.d. | M | 1 |
|  | *Urtica australis* Hook.f. | 0 | 0 | 0 | 0 | 1 | 1 | 0 | 1 | 0 | 0 | 0 | 1 | ab | n.d. | M | 14 |
| Violaceae | *Viola maculata* Cav. | 0 | 0 | 0 | 0 | 0 | 0 | 1 | 0 | 0 | 0 | 0 | 0 | b | SC | H | 5 |
|  | *Viola magellanica* Poir. | 0 | 0 | 0 | 0 | 0 | 0 | 1 | 0 | 0 | 0 | 0 | 0 | b | SC | H | 11 |
|  | *Viola tridentata* Sm. | 0 | 0 | 0 | 0 | 0 | 0 | 1 | 0 | 0 | 0 | 0 | 0 | b | SCp | H | 5 |

| **Monocots** |  |  |  |  |  |  |  |  |  |  |  |  |  |  |  |  |  |
| --- | --- | --- | --- | --- | --- | --- | --- | --- | --- | --- | --- | --- | --- | --- | --- | --- | --- |
| Alstroemeriaceae | *Luzuriaga marginata* (Gaertn.) Benth. & Hook.f. | 0 | 0 | 0 | 0 | 0 | 0 | 1 | 0 | 0 | 0 | 0 | 0 | B | n.d. | H | 5 |
| Asphodelaceae | *Bulbinella rossii* (Hook.f.) Cheeseman | 0 | 0 | 0 | 0 | 0 | 1 | 0 | 1 | 0 | 0 | 0 | 1 | B | - | D | 6, 42 |
| Asteliaceae | *Astelia linearis* Hook.f. | 0 | 0 | 0 | 0 | 0 | 1 | 0 | 0 | 0 | 0 | 0 | 0 | B | - | D | 6 |
|  | *Astelia pumila* (J.R.Forst.) Gaudich. | 0 | 0 | 0 | 0 | 0 | 0 | 1 | 0 | 0 | 0 | 0 | 0 | B | - | D | 5 |
|  | *Astelia subulata* (Hook.f.) Cheeseman | 0 | 0 | 0 | 0 | 0 | 1 | 0 | 1 | 0 | 0 | 0 | 0 | B | - | D | 6 |
| Centrolepidaceae | *Centrolepis ciliata* (Hook.f.) Druce | 0 | 0 | 0 | 0 | 0 | 1 | 0 | 1 | 0 | 0 | 0 | 0 | A | n.d. | M | 6 |
|  | *Centrolepis pallida* (Hook.f) Cheesem. | 0 | 0 | 0 | 0 | 0 | 1 | 0 | 1 | 0 | 0 | 0 | 0 | A | n.d. | M | 6 |
|  | *Gaimardia australis* Gaudich. | 0 | 0 | 0 | 0 | 0 | 0 | 1 | 0 | 0 | 0 | 0 | 0 | A | n.d. | M | 5 |
| Corsiaceae | *Arachnitis uniflora* Phil. | 0 | 0 | 0 | 0 | 0 | 0 | 1 | 0 | 0 | 0 | 0 | 0 | B | n.d. | H | 21 |
| Cyperaceae | *Carex acaulis* d'Urv. | 0 | 0 | 0 | 0 | 0 | 0 | 1 | 0 | 0 | 0 | 0 | 0 | A | n.d. | M | 5 |
|  | *Carex aematorrhychna* var.*corralensis* (Phil.) Kük | 0 | 0 | 0 | 0 | 0 | 0 | 1 | 0 | 0 | 0 | 0 | 0 | A | n.d. | M | 5 |
|  | *Carex appressa* R.Br. | 0 | 0 | 0 | 0 | 1 | 1 | 0 | 1 | 0 | 0 | 0 | 0 | A | n.d. | M | 6 |
|  | *Carex banksii* Boott | 0 | 0 | 0 | 0 | 0 | 0 | 1 | 0 | 0 | 0 | 0 | 0 | A | n.d. | M | 11 |
|  | *Carex caduca* Boott | 0 | 0 | 0 | 0 | 0 | 0 | 1 | 0 | 0 | 0 | 0 | 0 | A | n.d. | M | 5 |
|  | *Carex canescens* L. | 0 | 0 | 0 | 0 | 0 | 0 | 1 | 0 | 0 | 0 | 0 | 0 | A | n.d. | M | 5 |
|  | *Carex decidua* Boott | 0 | 0 | 0 | 0 | 0 | 0 | 1 | 0 | 0 | 0 | 0 | 0 | A | n.d. | M | 5 |
|  | *Carex fuscula* d'Urv. | 0 | 0 | 0 | 0 | 0 | 0 | 1 | 0 | 0 | 0 | 0 | 0 | A | n.d. | M | 5 |
|  | *Carex macloviana* d'Urv. | 0 | 0 | 0 | 0 | 0 | 0 | 1 | 0 | 0 | 0 | 0 | 0 | A | n.d. | M | 5 |
|  | *Carex magellanica* Lam. | 0 | 0 | 0 | 0 | 0 | 0 | 1 | 0 | 0 | 0 | 0 | 0 | A | n.d. | M | 5 |
|  | *Carex microglochin* subsp. *fuegina* Kük. | 0 | 0 | 0 | 0 | 0 | 0 | 1 | 0 | 0 | 0 | 0 | 0 | A | n.d. | M | 5 |
|  | *Carex sagei* Phil. | 0 | 0 | 0 | 0 | 0 | 0 | 1 | 0 | 0 | 0 | 0 | 0 | A | n.d. | M | 40 |
|  | *Carex sectoides* (Kük.) Edgar | 0 | 0 | 0 | 0 | 1 | 0 | 0 | 0 | 0 | 0 | 0 | 0 | A | n.d. | M | 6 |
|  | *Carex ternaria* G.Forst. | 0 | 0 | 0 | 0 | 1 | 1 | 0 | 0 | 0 | 0 | 0 | 0 | A | n.d. | M | 6 |
|  | *Carex trifida* Cav. | 0 | 0 | 1 | 0 | 1 | 1 | 0 | 1 | 0 | 0 | 1 | 0 | A | n.d. | M | 5, 6 |
|  | *Carex vallis-pulchrae* Phil. | 0 | 0 | 0 | 0 | 0 | 0 | 1 | 0 | 0 | 0 | 0 | 0 | A | n.d. | M | 5 |
|  | *Carpha alpina* R.Br. | 0 | 0 | 0 | 0 | 0 | 1 | 0 | 0 | 0 | 0 | 0 | 0 | A | n.d. | H | 6 |
|  | *Eleocharis melanostachys* (d'Urv.) C.B.Clarke | 0 | 0 | 0 | 0 | 0 | 0 | 1 | 0 | 0 | 0 | 0 | 0 | A | n.d. | H | 5 |
|  | *Isolepis aucklandica* Hook.f. | 0 | 0 | 0 | 0 | 1 | 1 | 0 | 1 | 0 | 0 | 1 | 0 | A | n.d. | H | 6 |
|  | *Isolepis cernua* (Vahl) Roem. & Schult. | 0 | 0 | 1 | 0 | 1 | 1 | 1 | 1 | 0 | 0 | 0 | 0 | A | n.d. | H | 5 |
|  | *Isolepis habra* (Edgar) Soják | 0 | 0 | 0 | 0 | 0 | 1 | 0 | 1 | 0 | 0 | 0 | 0 | A | n.d. | H | 6 |
|  | *Isolepis praetextata* (Edgar) Soják | 0 | 0 | 0 | 0 | 0 | 1 | 0 | 1 | 0 | 0 | 0 | 0 | A | n.d. | H | 6 |
|  | *Oreobolus obtusangulus* Gaudich. | 0 | 0 | 0 | 0 | 0 | 0 | 1 | 0 | 0 | 0 | 0 | 0 | A | n.d. | H | 5 |
|  | *Oreobolus pectinatus* Hook.f. | 0 | 0 | 0 | 0 | 0 | 1 | 0 | 1 | 0 | 0 | 0 | 0 | A | n.d. | H | 6 |
|  | *Schoenoplectus californicus* (C.A.Mey.) Soják | 0 | 0 | 0 | 0 | 0 | 0 | 1 | 0 | 0 | 0 | 0 | 0 | A | n.d. | H | 5 |
|  | *Schoenus pauciflorus* (Hook.f.) Hook.f. | 0 | 0 | 0 | 0 | 0 | 1 | 0 | 0 | 0 | 0 | 0 | 0 | A | n.d. | H | 6 |
|  | *Uncinia aucklandica* Hamlin | 0 | 0 | 0 | 0 | 0 | 1 | 0 | 1 | 0 | 0 | 0 | 0 | A | n.d. | M | 6 |
|  | *Uncinia compacta* R.Br. | 1 | 1 | 0 | 1 | 0 | 0 | 0 | 0 | 0 | 0 | 0 | 1 | A | n.d. | M | 6 |
|  | *Uncinia divaricata* Boott | 0 | 0 | 0 | 0 | 0 | 0 | 0 | 1 | 0 | 0 | 1 | 0 | A | n.d. | M | 6 |
|  | *Uncinia hookeri* Boott | 0 | 0 | 0 | 0 | 1 | 1 | 0 | 1 | 0 | 0 | 1 | 1 | A | n.d. | M | 6 |
|  | *Uncinia macloviana* Gaudich. | 0 | 0 | 0 | 0 | 0 | 0 | 1 | 0 | 0 | 0 | 0 | 0 | A | n.d. | M | 5 |
|  | *Uncinia macrolepis* Decne. | 0 | 0 | 0 | 0 | 0 | 0 | 0 | 0 | 0 | 1 | 0 | 0 | A | n.d. | M | 2, 10 |
|  | *Uncinia strictissima* (Kük.) Petrie | 0 | 0 | 0 | 0 | 0 | 1 | 0 | 0 | 0 | 0 | 0 | 0 | A | n.d. | M | 6 |
|  | *Uncinia uncinata* (L.f.) Kük. | 0 | 0 | 0 | 0 | 0 | 1 | 0 | 0 | 0 | 0 | 0 | 0 | A | n.d. | M | 6 |
| Iridaceae | *Olsynium filifolium* (Gaudich.) Goldblatt | 0 | 0 | 0 | 0 | 0 | 0 | 1 | 0 | 0 | 0 | 0 | 1 | B | n.d. | H | 5, 17 |
|  | *Sisyrinchium chilense* Hook. | 0 | 0 | 0 | 0 | 0 | 0 | 1 | 0 | 0 | 0 | 0 | 0 | B | n.d. | H | 5 |
| Juncaceae | *Juncus antarcticus* Hook.f. | 0 | 0 | 0 | 0 | 0 | 1 | 0 | 1 | 0 | 0 | 0 | 0 | A | n.d. | H | 6 |
|  | *Juncus pusillus* Buchenau | 1 | 0 | 0 | 1 | 0 | 1 | 0 | 0 | 0 | 0 | 0 | 0 | A | n.d. | H | 6 |
|  | *Juncus scheuchzerioides* Gaudich. | 1 | 1 | 0 | 1 | 1 | 1 | 1 | 1 | 0 | 1 | 1 | 0 | A | SC | H | 2, 5, 10 |
|  | *Luzula alopecurus* Desv. | 0 | 0 | 0 | 0 | 0 | 0 | 1 | 0 | 0 | 0 | 0 | 0 | A | SC | H | 5 |
|  | *Luzula banksiana* E.Mey. | 0 | 0 | 0 | 0 | 0 | 1 | 0 | 0 | 0 | 0 | 0 | 0 | A | n.d. | H | 6 |
|  | *Luzula crinita* Hook.f. | 0 | 0 | 0 | 0 | 1 | 1 | 0 | 1 | 0 | 0 | 1 | 0 | A | n.d. | H | 6 |
|  | *Marsippospermum gracile* (Hook.f.) Buchenau | 0 | 0 | 0 | 0 | 0 | 1 | 0 | 1 | 0 | 0 | 0 | 0 | A | n.d. | H | 6 |
|  | *Marsippospermum grandiflorum* (L.f.) Hook. | 0 | 0 | 0 | 0 | 0 | 0 | 1 | 0 | 0 | 0 | 0 | 0 | A | n.d. | H | 5 |
|  | *Rostkovia magellanica* (Lam.) Hook.f. | 0 | 0 | 0 | 0 | 0 | 1 | 1 | 1 | 0 | 1 | 0 | 0 | A | n.d. | H | 2, 5, 10 |
| Juncaginaceae | *Tetroncium magellanicum* Willd. | 0 | 0 | 0 | 0 | 0 | 0 | 1 | 0 | 0 | 0 | 0 | 0 | A | - | D | 5 |
|  | *Triglochin striata* Ruiz & Pav. | 0 | 0 | 0 | 0 | 0 | 1 | 0 | 0 | 0 | 0 | 0 | 0 | A | n.d. | H | 6 |
| Orchidaceae | *Aporostylis bifolia* (Hook.f.) Rupp & Hatch | 0 | 0 | 0 | 0 | 1 | 1 | 0 | 1 | 0 | 0 | 0 | 0 | B | n.d. | H | 6 |
|  | *Caladenia carnea* R.Br. | 0 | 0 | 0 | 0 | 0 | 1 | 0 | 0 | 0 | 0 | 0 | 0 | B | n.d. | H | 6 |
|  | *Chiloglottis cornuta* Hook.f. | 0 | 0 | 0 | 0 | 1 | 1 | 0 | 1 | 0 | 0 | 0 | 0 | B | SC | H | 14 |
|  | *Chloraea fonkii* Phil. | 0 | 0 | 0 | 0 | 0 | 0 | 1 | 0 | 0 | 0 | 0 | 0 | B | n.d. | H | 5 |
|  | *Codonorchis lessonii* (d'Urv.) Lindl. | 0 | 0 | 0 | 0 | 0 | 0 | 1 | 0 | 0 | 0 | 0 | 0 | B | n.d. | H | 5 |
|  | *Gavilea australis* (Skottsb.) M.N.Correa | 0 | 0 | 0 | 0 | 0 | 0 | 1 | 0 | 0 | 0 | 0 | 0 | B | n.d. | H | 5 |
|  | *Gavilea littoralis* (Phil.) M.N.Correa | 0 | 0 | 0 | 0 | 0 | 0 | 1 | 0 | 0 | 0 | 0 | 0 | B | n.d. | H | 5 |
|  | *Nematoceras dienemum* (D.L.Jones) D.L.Jones,M.A.Clem. & Molloy | 0 | 0 | 0 | 0 | 0 | 0 | 0 | 0 | 0 | 0 | 1 | 1 | B | n.d. | H | 29 |
|  | *Nematoceras macranthum* Hook.f. | 0 | 0 | 0 | 0 | 0 | 1 | 0 | 0 | 0 | 0 | 0 | 0 | B | n.d. | H | 6 |
|  | *Nematoceras rivulare* (A.Cunn.) Hook.f. | 0 | 0 | 0 | 0 | 0 | 1 | 0 | 0 | 0 | 0 | 0 | 0 | B | n.d. | H | 6 |
|  | *Nematoceras sulcatum* M.A.Clem & D.L.Jones | 0 | 0 | 0 | 0 | 0 | 0 | 0 | 0 | 0 | 0 | 1 | 1 | B | n.d. | H | 29 |
|  | *Nematoceras trilobum* Hook.f. | 0 | 0 | 0 | 0 | 1 | 1 | 0 | 1 | 0 | 0 | 0 | 0 | B | n.d. | H | 6 |
|  | *Prasophyllum colensoi* Hook.f. | 0 | 0 | 0 | 0 | 1 | 1 | 0 | 0 | 0 | 0 | 0 | 0 | B | n.d. | H | 6 |
|  | *Singlarybas oblongus* (Hook.f.) Mollay,D.L.Jones & M.A. Clem. | 0 | 0 | 0 | 0 | 0 | 1 | 0 | 0 | 0 | 0 | 0 | 0 | B | n.d. | H | 6 |
|  | *Thelymitra cyanea* R.Br. | 0 | 0 | 0 | 0 | 0 | 1 | 0 | 0 | 0 | 0 | 0 | 0 | B | n.d. | H | 6 |
|  | *Thelymitra longifolia* J.R.Forst. & G.Forst. | 0 | 0 | 0 | 0 | 0 | 1 | 0 | 0 | 0 | 0 | 0 | 0 | B | SC | H | 27 |
|  | *Townsonia deflexa* (Hook.f.) Schltr. | 0 | 0 | 0 | 0 | 0 | 1 | 0 | 1 | 0 | 0 | 0 | 0 | B | n.d. | H | 6 |
|  | *Waireia stenopetala* (Hook.f.) D.L.Jones, M.A.Clem. & Molloy | 0 | 0 | 0 | 0 | 0 | 1 | 0 | 1 | 0 | 0 | 0 | 0 | B | n.d. | H | 6 |
| Poaceae | *Agrostis magellanica* Lam. | 1 | 1 | 0 | 1 | 1 | 1 | 1 | 1 | 0 | 0 | 0 | 0 | A | n.d. | H | 5 |
|  | *Agrostis meyenii* Trin. | 0 | 0 | 0 | 0 | 0 | 0 | 1 | 0 | 0 | 0 | 0 | 0 | A | n.d. | H | 5 |
|  | *Agrostis subulata* Hook. F. | 0 | 0 | 0 | 0 | 0 | 1 | 0 | 1 | 0 | 0 | 0 | 1 | A | n.d. | H | 23 |
|  | *Alopecurus magellanicus* Lam. | 0 | 0 | 0 | 0 | 0 | 0 | 1 | 0 | 0 | 1 | 0 | 0 | A | n.d. | H | 2, 5 |
|  | *Chionochloa antarctica* (Hook.f.) Zotov | 0 | 0 | 0 | 0 | 0 | 1 | 0 | 1 | 0 | 0 | 0 | 1 | A | n.d. | H | 23 |
|  | *Cortaderia pilosa* (d'Urv.) Hack. | 0 | 0 | 0 | 0 | 0 | 0 | 1 | 0 | 0 | 0 | 0 | 0 | A | n.d. | GD | 5 |
|  | *Deschampsia antarctica* É.Desv. | 1 | 0 | 0 | 1 | 0 | 0 | 1 | 0 | 1 | 1 | 0 | 0 | A | SC | H | 2, 5, 10 |
|  | *Deschampsia cespitosa* (L.) Beauv. | 0 | 0 | 0 | 0 | 0 | 0 | 0 | 0 | 0 | 0 | 1 | 0 | A | n.d. | H | 23 |
|  | *Deschampsia chapmanii* Petrie | 0 | 0 | 0 | 0 | 0 | 1 | 0 | 1 | 0 | 0 | 1 | 0 | A | n.d. | H | 23 |
|  | *Deschampsia flexuosa* (L.) Trin. | 0 | 0 | 0 | 0 | 0 | 0 | 1 | 0 | 0 | 0 | 0 | 0 | A | n.d. | H | 5 |
|  | *Deschampsia gracillima* Kirk | 0 | 0 | 0 | 0 | 0 | 1 | 0 | 1 | 0 | 0 | 0 | 0 | A | n.d. | H | 23 |
|  | *Deschampsia parvula* (Hook.f.) É.Desv. | 0 | 0 | 0 | 0 | 0 | 0 | 1 | 0 | 0 | 0 | 0 | 0 | A | n.d. | H | 5 |
|  | *Deyeuxia aucklandica* (Hook.f.) Zotov | 0 | 0 | 0 | 0 | 0 | 1 | 0 | 0 | 0 | 0 | 0 | 0 | A | n.d. | H | 23 |
|  | *Elymus magellanicus* (Desv.) Á.Löve | 0 | 0 | 0 | 0 | 0 | 0 | 1 | 0 | 0 | 0 | 0 | 0 | A | n.d. | H | 23 |
|  | *Festuca contracta* Kirk | 0 | 0 | 0 | 1 | 0 | 0 | 1 | 0 | 0 | 1 | 1 | 1 | A | n.d. | H | 2, 5, 10 |
|  | *Festuca magellanica* Lam. | 0 | 0 | 0 | 0 | 0 | 0 | 1 | 0 | 0 | 0 | 0 | 0 | A | n.d. | H | 5 |
|  | *Hierochloe brunonis* Hook.f. | 0 | 0 | 0 | 0 | 0 | 1 | 0 | 1 | 0 | 0 | 0 | 0 | A | n.d. | AM | 23 |
|  | *Hierochloe fusca* Zotov | 0 | 0 | 0 | 0 | 0 | 1 | 0 | 1 | 0 | 0 | 0 | 0 | A | n.d. | AM | 23 |
|  | *Hierochloe redolens* (Vahl) Roem. & Schult. | 0 | 0 | 0 | 0 | 0 | 0 | 1 | 1 | 0 | 0 | 0 | 0 | A | n.d. | AM | 5 |
|  | *Koeleria permollis* Steud. | 0 | 0 | 0 | 0 | 0 | 0 | 1 | 0 | 0 | 0 | 0 | 0 | A | n.d. | H | 5 |
|  | *Lachnagrostis leptostachys* (Hook.f.) Zotov | 0 | 0 | 0 | 0 | 1 | 1 | 0 | 1 | 0 | 0 | 0 | 1 | A | n.d. | H | 23 |
|  | *Lachnagrostis pilosa* (Buchanan) Edgar | 0 | 0 | 0 | 0 | 0 | 1 | 0 | 0 | 0 | 0 | 0 | 0 | A | n.d. | H | 23 |
|  | *Microlaena avenacea* (Raoul) Hook.f. | 0 | 0 | 0 | 0 | 0 | 1 | 0 | 0 | 0 | 0 | 0 | 0 | A | n.d. | H | 23 |
|  | *Phleum alpinum* L. | 0 | 0 | 0 | 0 | 0 | 0 | 0 | 0 | 0 | 1 | 0 | 0 | A | n.d. | H | 10 |
|  | *Poa alopecurus* (Gaudich.) Kunth | 0 | 0 | 0 | 0 | 0 | 0 | 1 | 0 | 0 | 0 | 0 | 0 | A | - | D | 5 |
|  | *Poa antipoda* Petrie | 0 | 0 | 0 | 0 | 1 | 1 | 0 | 1 | 0 | 0 | 0 | 0 | A | n.d. | GM / H | 23 |
|  | *Poa astonii* Petrie | 0 | 0 | 1 | 0 | 0 | 1 | 0 | 0 | 0 | 0 | 0 | 0 | A | n.d. | H | 23 |
|  | *Poa aucklandica* Petrie | 0 | 0 | 0 | 0 | 0 | 1 | 0 | 0 | 0 | 0 | 0 | 0 | A | n.d. | H | 23 |
|  | *Poa aucklandica* subsp. *campbellensis* (Petrie) Edgar | 0 | 0 | 0 | 0 | 0 | 0 | 0 | 1 | 0 | 0 | 0 | 0 | A | n.d. | H | 23 |
|  | *Poa breviglumis* Hook.f. | 0 | 0 | 0 | 0 | 1 | 1 | 0 | 1 | 0 | 0 | 0 | 0 | A | SC | H | 14 |
|  | *Poa cookii* (Hook.f.) Hook.f. | 1 | 1 | 0 | 1 | 0 | 0 | 0 | 0 | 1 | 0 | 1 | 1 | A | n.d. | GM | 23 |
|  | *Poa flabellata* (Lam.) Raspail | 0 | 0 | 0 | 0 | 0 | 0 | 1 | 0 | 0 | 1 | 0 | 0 | A | n.d. | H | 2, 5, 10 |
|  | *Poa foliosa* (Hook.f.) Hook.f. | 0 | 0 | 0 | 0 | 1 | 1 | 0 | 1 | 0 | 0 | 1 | 0 | A | - | D | 23 |
|  | *Poa incrassata* Petrie | 0 | 0 | 0 | 0 | 0 | 1 | 0 | 0 | 0 | 0 | 0 | 0 | A | n.d. | H | 23 |
|  | *Poa kerguelensis* (Hook.f.) Steud. | 0 | 0 | 0 | 1 | 0 | 0 | 0 | 0 | 1 | 0 | 0 | 1 | A | n.d. | H | 19 |
|  | *Poa litorosa* Cheeseman | 0 | 0 | 0 | 0 | 1 | 1 | 0 | 1 | 0 | 0 | 1 | 1 | A | n.d. | H | 23 |
|  | *Poa novae-zelandiae* Hack. | 0 | 0 | 0 | 0 | 0 | 1 | 0 | 0 | 0 | 0 | 0 | 0 | A | n.d. | GM | 23 |
|  | *Poa ramosissima* Hook.f. | 0 | 0 | 0 | 0 | 0 | 1 | 0 | 1 | 0 | 0 | 0 | 1 | A | n.d. | GM | 23 |
|  | *Poa robusta* Steud. | 0 | 0 | 0 | 0 | 0 | 0 | 1 | 0 | 0 | 0 | 0 | 0 | A | n.d. | GD | 5 |
|  | *Poa tennantiana* Petrie | 0 | 0 | 1 | 0 | 0 | 1 | 0 | 0 | 0 | 0 | 0 | 0 | A | n.d. | H | 23 |
|  | *Puccinellia antipoda* (Petrie) Allan & Jansen | 0 | 0 | 0 | 0 | 1 | 0 | 0 | 0 | 0 | 0 | 0 | 1 | A | SC | H | 23 |
|  | *Puccinellia chathamica* (Cheeseman) Allan & Jansen | 0 | 0 | 0 | 0 | 0 | 1 | 0 | 1 | 0 | 0 | 0 | 0 | A | SC | H | 23 |
|  | *Puccinellia macquariensis* (Cheeseman) Allan & Jansen | 0 | 0 | 0 | 0 | 0 | 0 | 0 | 0 | 0 | 0 | 1 | 1 | A | SC | H | 23 |
|  | *Puccinellia pusilla* (Hack.) Parodi | 0 | 0 | 0 | 0 | 0 | 0 | 1 | 0 | 0 | 0 | 0 | 0 | A | n.d. | H | 5 |
|  | *Trisetum phleoides* (d'Urv.) Kunth | 0 | 0 | 0 | 0 | 0 | 0 | 1 | 0 | 0 | 0 | 0 | 0 | A | n.d. | H | 5 |
|  | *Trisetum spicatum* (L.) K.Richt. | 0 | 0 | 0 | 0 | 0 | 1 | 0 | 1 | 0 | 0 | 0 | 0 | A | n.d. | H | 23 |
|  | *Zotovia thomsonii* (Petrie) Edgar & Connor | 0 | 0 | 0 | 0 | 0 | 1 | 0 | 0 | 0 | 0 | 0 | 0 | A | n.d. | H | 23 |

**References**

Arroyo MTK, von Bohlen CP, Cavieres L, Marticorena C. 1992. Survey of the alpine flora of Torres del Paine National Park, Chile. *Gayana Botanica* **49**:47–70.

Bischoff M. 2008. Pollination ecology of the New Zealand alpine flora. PhD Thesis, University of Heidelberg, Germany.

Cerfonteyn ME, Le Roux PC, Van Vuuren BJ, Born C. 2011. Cryptic spatial aggregation of the cushion plant Azorella selago (Apiaceae) revealed by a multilocus molecular approach suggests frequent intraspecific facilitation under sub-Antarctic conditions. *American Journal of Botany* **98**:909–914.

Clements MA, MacKenzie AM, Copson GR, Molloy BPJ, Carmichael N, Skotnicki ML, Selkirk PM. 2007. Biology and molecular phylogenetics of Nematoceras sulcatum, a second endemic orchid species from subantarctic Macquarie Island. *Polar Biology* **30**: 859–869.

Glenny D. 2004. A revision of the genus Gentianella in New Zealand. *New Zealand Journal of Botany* **42**:361–530.

Godley EJ. 1966. Breeding systems in NewZealand plants. 4. Self-sterility in Pentachondra pumila. *New Zealand Journal of Botany* **4**:249–254.

Godley EJ. 1985. A visit to the Auckland islands in the summer of 1962–63. *Tuatara* **28**:1–12.

Goldblatt P, Rudall P, Henrich JE. 1990. The genera of the Sisyrinchium alliance (Iridaceae: Iridoideae): phylogeny and relationships. *Systematic Botany* **15**:497–510.

Heenan PB. 2008. Cardamine latior (Brassicaceae), a new species endemic to the subantarctic Auckland Islands, New Zealand. *New Zealand Journal of Botany* **46**:559–566.

Ibisch PL, Neinhuis C, Rojas P. 1996. On the biology, biogeography, and taxonomy of Arachnitis Phil. Nom. Cons. (Corsiaceae) in respect to a new record from Bolivia. *Wildenowia* **26**:321–332.

Lehnebach CA, Robertson AW, Hedderley D. 2005. Pollination studies of four New Zealand terrestrial orchids and the implication for their conservation. *New Zealand Journal of Botany* **43**:467–477.

Lloyd DG. 1972. A revision of the New Zealand, Subantarctic, and South American species of Cotula, section Leptinella. *New Zealand Journal of Botany* **10**:277–372.

Lord JM. 2012. Hermaphroditism and dichogamy in Stilbocarpa polaris (Araliaceae) on Campbell Island. *New Zealand Journal of Botany* **50**:89–93.

Martinez S. 1993. Sinopsis del gene´ro Azorella (Apiaceae, Hydrocotyloidae). *Darwinia* **32**:171–184.

Nilsson E, Ågren J. 2006. Population size, female fecundity, and sex ratio variation in gynodioecious Plantago maritima. *Journal of Evolutionary Biology* **19**:825–833.

Orchard AE. 1989. Azorella Lamark (Apiaceae) on Heard and Macquarie islands, with descriptions of a new species, *A. macquariensis. Muelleria* **7**:15–20.

Percival M. 1965. *Floral biology*. Oxford, UK: Pergamon Press.

Raven PH, Raven TM. 1976. *The genus Epilobium(Onagraceae) in Australasia: a systematic and evolutionary study.* New Zealand DSIR Bulletin 216. Christchurch: Government Printer.

Rendle H, Murray BG. 1988. Breeding systems and pollen tube behaviour in compatible and incompatible crosses in New Zealand species of Ranunculus L*. New Zealand Journal of Botany* **26**:467–471.

Sciligo AR. 2009. Food or sex: which would you choose? Pollinatorprey conflict and reproductive assurance in New Zealand Drosera. PhD Thesis, Lincoln University, New Zealand.

Sersic AN. 2004. *Pollination biology in the genus Calceolaria L.* (*Calceolariaceae*)*.* Biology Centre of the Upper Austrian Museums.

Walton DWH. 1979. Studies on Acaena (Rosaceae): III. Flowering and hybridisation on South Georgia. *British Antarctic Survey Bulletin* **48**:1–13.

Zavala-Gallo L, Denham S, Pozner R. 2010. Revision of the genus Nastanthus (Calyceraceae)*. Gayana Botánica* **67**:158–175.
